# Impacts of climate change on species distribution patterns of *Polyspora* sweet in China

**DOI:** 10.1002/ece3.9516

**Published:** 2022-12-13

**Authors:** Zhi‐Feng Fan, Bing‐Jiang Zhou, Chang‐Le Ma, Can Gao, Dan‐Ni Han, Yong Chai

**Affiliations:** ^1^ Southwest Research Center for Engineering Technology of Landscape Architecture (State Forestry and Grassland Administration), College of Landscape Architecture and Horticulture Sciences Southwest Forestry University Kunming China; ^2^ Kunming University of Science and Technology Kunming China; ^3^ Experimental Center of Tropical Forestry Chinese Academy of Forestry Pingxiang China; ^4^ Yunnan Academy of Forestry and Grassland Kunming China

**Keywords:** climate change, genus *Polyspora*, glacial refugia, MaxEnt model, potential suitable areas

## Abstract

Climate change is an important driver of species distribution and biodiversity. Understanding the response of plants to climate change is helpful to understand species differentiation and formulate conservation strategies. The genus *Polyspora* (Theaceae) has an ancient origin and is widely distributed in subtropical evergreen broad‐leaved forests. Studies on the impacts of climate change on species geographical distribution of Chinese *Polyspora* can provide an important reference for exploring the responses of plant groups in subtropical evergreen broad‐leaved forests with geological events and climate change in China. Based on the environmental variables, distribution records, and chloroplast genomes, we modeled the potential distribution of Chinese *Polyspora* in the Last Glacial Maximum, middle Holocene, current, and future by using MaxEnt‐ArcGIS model and molecular phylogenetic method. The changes in the species distribution area, centroid shift, and ecological niche in each periods were analyzed to speculate the response modes of Chinese *Polyspora* to climate change in different periods. The most important environmental factor affecting the distribution of *Polyspora* was the precipitation of the driest month, ranging from 13 to 25 mm for the highly suitable habitats. At present, highly suitable distribution areas of *Polyspora* were mainly located in the south of 25°N, and had species‐specificity. The main glacial refugia of the Chinese *Polyspora* might be located in the Ailao, Gaoligong, and Dawei Mountains of Yunnan Province. Jinping County, Pingbian County, and the Maguan County at the border of China and Vietnam might be the species differentiation center of the Chinese *Polyspora*. Moderate climate warming in the future would be beneficial to the survival of *P. axillaris*, *P. chrysandra*, and *P. speciosa*. However, climate warming under different shared socio‐economic pathways would reduce the suitable habitats of *P. hainanensis* and *P. longicarpa*.

## Graphical abstract

During the LGM 22,000 years ago, there were overlapping distributions of various types and regions of Chinese *Polyspora* species. Jinping County, Pingbian County, and the Maguan County at the border of China and Vietnam might be the species differentiation center of the Chinese *Polyspora*.

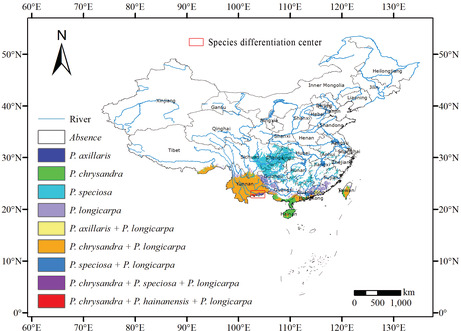

## 1 INTRODUCTION

Climate change is one of the most important natural factors affecting biodiversity and species distribution (Araujo & Rahbek, 2006; Lenoir et al., 2008; Thuiller, 2007). The drastic climatic changes during the Quaternary glaciation had a profound impact on the geographical distribution patterns and species differentiation (Comes & Kadereit, 1998; Gao & Zhao, 2021; Sandel et al., 2011). The Last Glacial Maximum (LGM, 22 ka) was one of the coldest and driest periods in Earth's history (Clark et al., 2009). The glacial period led to the extinction of some terrestrial species, and some species evolved adaptively in situ or retreated to refugia (Jackson & Overpeck, 2000; Nogues‐Bravo et al., 2010). As the climate warmed after the glacial periods, the surviving plants migrate and spread from the refugia to new habitats (Davis & Shaw, 2001; Normand et al., 2011). East Asia was not completely covered by ice and snow during the LGM, and the plants may have experienced a more complicated history (Hewitt, 2000). Reconstructing historical distribution dynamics of species would help us better understand the response of species to climate change and the historical causes of species differentiation and formation (Bai et al., 2018; Provan & Bennett, 2008).

With global warming, climate change will surpass habitat destruction and become the biggest threat to biodiversity in the future, which will have varying degrees of impact on biological individuals, populations, communities, and ecosystems (Bellard et al., 2012). According to the sixth assessment report of the Intergovernmental Panel on Climate Change (IPCC), the global average surface temperature in 2011–2020 was 1.09°C warmer than in 1850–1900; Over the next 20 years, global warming is expected to reach or exceed 1.5°C; By the end of the century, the global mean surface temperature will increase by 1.5–5.1°C (IPCC, 2021). Existing studies have shown that climate warming will reduce the suitable area of some plants and show a fragmented distribution (Meng et al., 2019; Qiu et al., 2020; Wan et al., 2021). In addition, climate change may also have a positive impact on biodiversity. Milder temperature and increased carbon dioxide may be beneficial to many plants, leading to accelerated biomass production. Warmer winters may increase the survival rate of many species, and increased precipitation will benefit some moisture‐loving plants (Bellard et al., 2012). Therefore, understanding the impact of future climate change on the distribution patterns of different species can provide a scientific basis for the formulation of species conservation strategies.

Niche model, also known as species distribution model, is a method of using known species distribution data and relevant environmental variables to build a model according to certain algorithm rules, judge the ecological needs of species, and project the calculation results to different time and space to predict the potential distribution area of species (Zhu et al., 2013). The development of niche model began with the development and application of BIOCLIM model (Booth et al., 2014). Subsequently, Ecological‐Niche Factor Analysis (ENFA), Maximum Entropy (MaxEnt), Generalized Linear Model (GLM), Generalized Addition Model (GAM), Classification and Regression Tree (CART), Multiple Adaptive Regression Spline (MARS), Genetic Algorithm for Rule‐set Prediction (GARP), Artificial Neural Networks (ANN), and other models (Xu et al., 2015). Among them, MaxEnt model has short operation time, stable operation results (Phillips et al., 2006) and high consistency between the predicted coverage and the actual distribution range of species (Booth et al., 2014; Ma et al., 2019). MaxEnt model is the most widely used niche model with good prediction effect (Giovanelli et al., 2008; Phillips & Dudik, 2008; Wang et al., 2020), which has been widely used in species evolution history (Jiang et al., 2016; Wang et al., 2021), impact of climate change on species distribution (Ren et al., 2020), endangered species protection (Wu et al., 2021), plant introduction and cultivation (Wang et al., 2021), and other fields.

**FIGURE 1:**
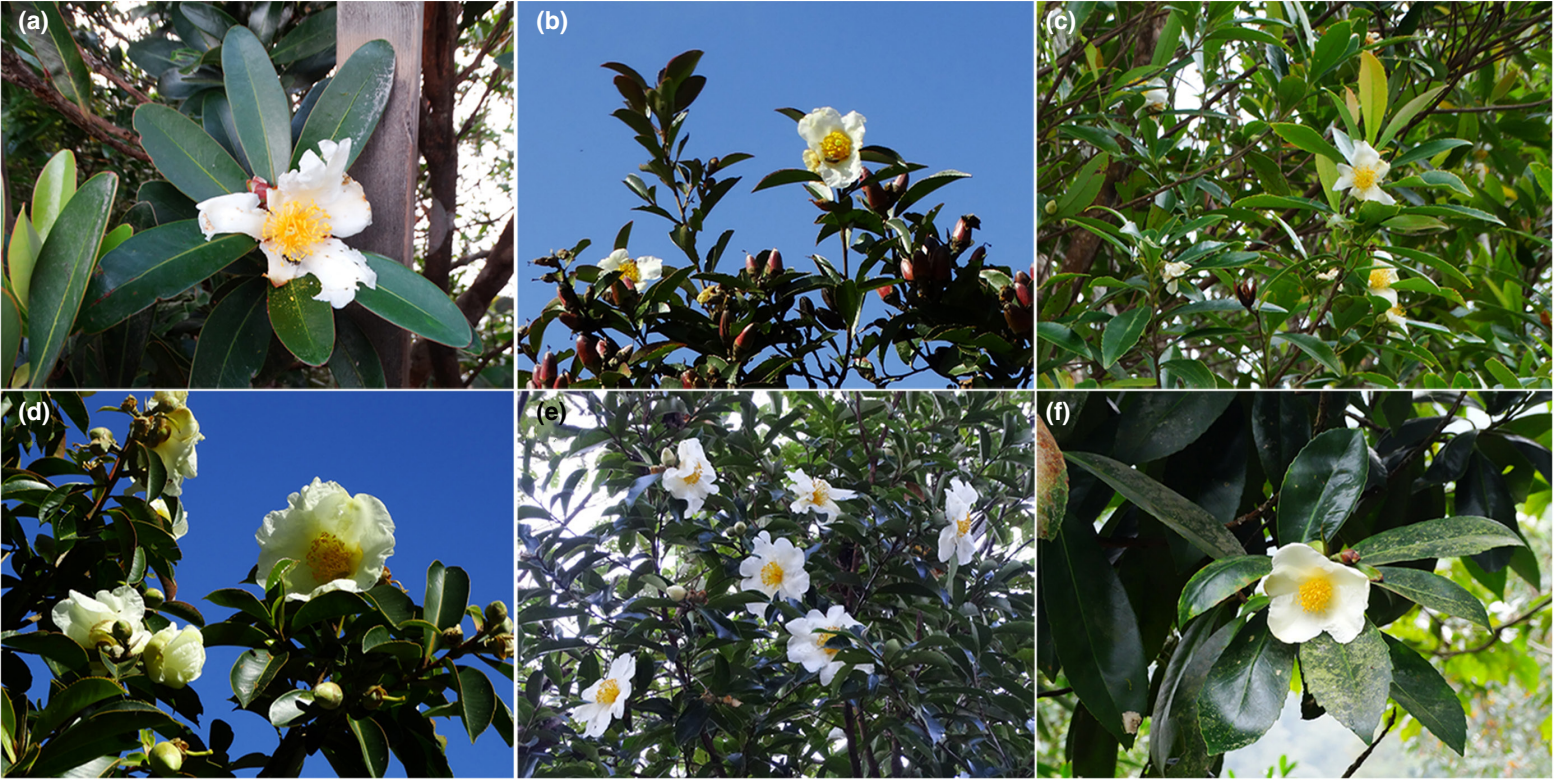
Photos of Chinese *Polyspora* species. (a) *Polyspora axillaris*; (b) *P. chrysandra*; (c) *P. hainanensis*; (d) *P. longicarpa*; (e) *P. tiantangensis*; and (f) *P. speciosa*. All the photos were taken by the first author.

*Polyspora* sweet belongs to Theaceae, species within the genus are evergreen tree or shrub. There are nearly 50 *Polyspora* species in the world, distributed in South and Southeast Asia, mainly in Malaysia, Indonesia, China, Vietnam, and other countries (Choo et al., 2020; Nguyet et al., 2020). *Polyspora* mainly grows in tropical and subtropical evergreen broad‐leaved forests. There are six species of *Polyspora* in China, including *P. axillaris*, *P. chrysandra*, *P. speciosa*, *P. hainanensis*, *P. longicarpa*, and *P. tiantangensis* (Figure 1), which are mainly distributed in southwest and south China (Ming & Bartholomew, 2007), growing in mountain forests or shrubland, ridges, valleys, and mountainsides. The genus *Polyspora* has high‐ornamental value, beautiful tree shape and blooming in winter. It can be used as shade tree and street tree in gardens (Fan, Han, et al., 2021; Fan, Qian, et al., 2021; Ma et al., 2015). Some species have edible and medicinal value, the fruits contain natural antioxidants (Li et al., 2017, 2019), and the extracts of roots and stems have cytotoxic activity (Fu, 2012; Tang, 2013; Xu et al., 2019). Previous studies have shown that environmental and evolutionary factors play important roles in shaping species richness patterns of Theaceae in China (Rao et al., 2018). *Polyspora* has an ancient origin and completed species differentiation in the late Pliocene (Zhang et al., 2022), and experienced the whole Quaternary climate change process. It is widely distributed and covers most of the subtropical areas in China, and is a typical representative in the subtropical evergreen broad‐leaved forest. Therefore, the study on the distribution dynamics of *Polyspora* in different historical periods can not only provide new clues for understanding the species evolution of subtropical evergreen broad‐leaved forest in China during the Quaternary ice age, but also provide scientific basis for the formulation of conservation measures and introduction and cultivation of *Polyspora* at present and in the future.

Based on the geographical distribution records and high‐resolution meteorological data of six Chinese *Polyspora* species, we used MaxEnt model and ArcGIS technology, combined with chloroplast genome data, to answer the following three questions: (1) What changes have taken place in the temporal and spatial distribution of *Polyspora* in China since the LGM period? Where are the current and future suitable distribution areas?; (2) Which are the key climatic factors affecting the distribution of *Polyspora*?; and (3) Where are the species differentiation centers and glacial refugia of *Polyspora* in China?

## 2 MATERIALS AND METHODS

### 2.1 Species distribution data

**FIGURE 2:**
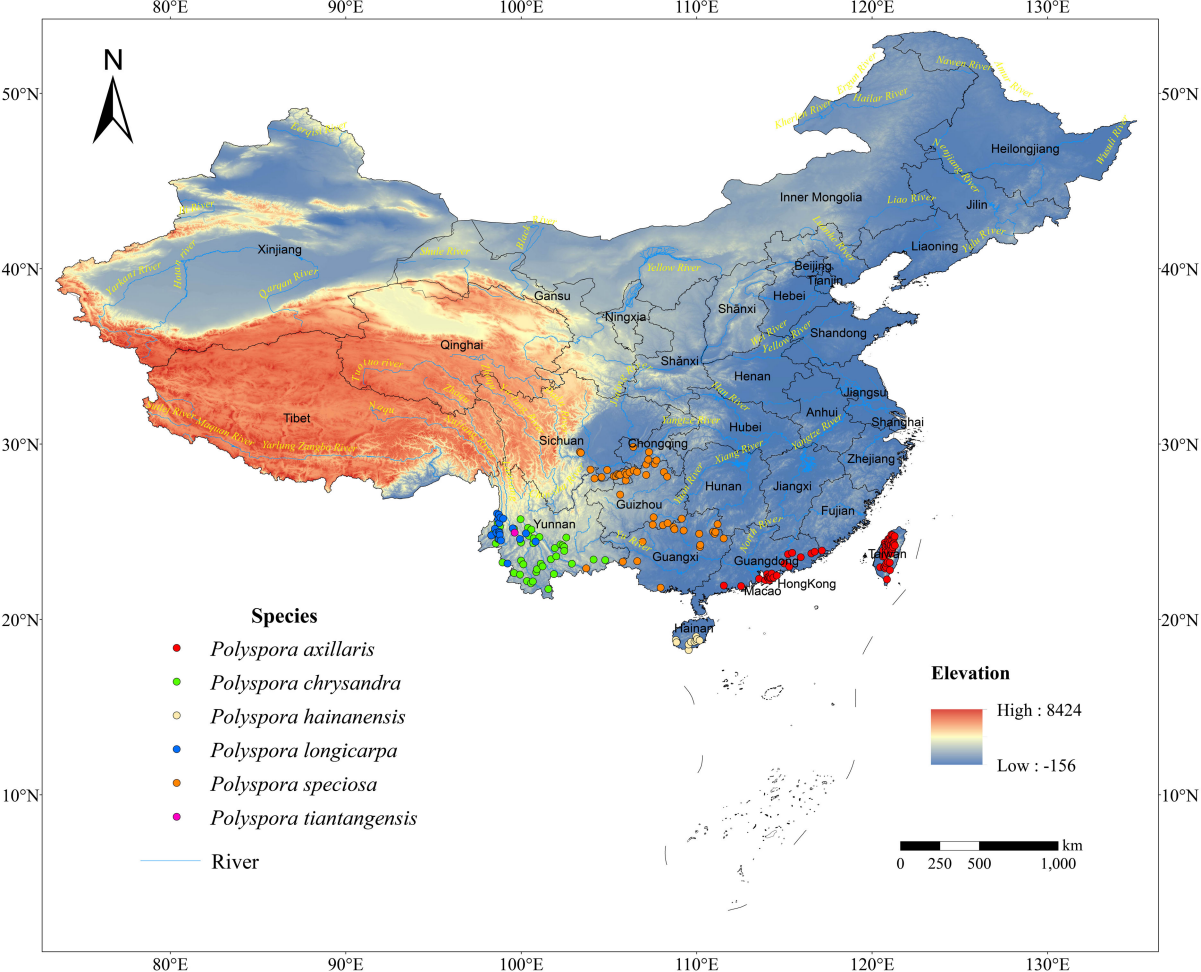
Occurrence points of *Polyspora* in China. The black font in the map refers to the names of provinces, and the yellow fonts refers to the names of rivers.

The geographical distribution data of *Polyspora* in China were mainly from the National Specimen Information Infrastructure (NSII, http://www.nsii.org.cn), Chinese Virtual Herbarium (CVH, https://www.cvh.ac.cn), Global Biodiversity Information Facility (GBIF, https://www.gbif.org), the published relevant literature, and field investigation from 2020 to 2022. For the sample points with a long history and no longitude or latitude information, Google Earth was used to locate and supplement them. Each specimen and each occurrence locality were carefully checked. The misidentified specimen and occurrences recorded outside the native range of the species in the GBIF were deleted. To reduce the spatial self‐correlation of population distribution points, avoid excessive fitting during MaxEnt operation, only one distribution point data was kept in the grid of 2.5 “× 2.5” on the map. Finally, in total 184 Chinese *Polyspora* occurrence sites were employed to build the model, including 63 *P. axillaris*, 43 *P. chrysandra*, 10 *P. hainanensis*, 19 *P. longicarpa*, 48 *P. speciosa*, and one *P. tiantangensis* (Table S1, Figure 2), these distribution records had covered the distribution range of each species. All the above, 184 distribution points were used in the prediction of the genus distribution area. According to unpublished data of our team, *P. tiantangensis* is actually an intraspecific variation type of *P. longicarpa*. In the simulation of species differentiation within the genus and the prediction at the species level, *P. tiantangensis* with only one distribution point was incorporated into *P. longicarpa*.

### 2.2 Environmental data

Environmental factors such as climate, ultraviolet radiation, soil and terrain affect the growth, development and reproduction of plants, and then determine their distribution. In this study, five types of environmental datasets were selected, with a total of 36 environmental variables, including 19 bioclimatic variables, six UV‐B variables, seven soil quality variables, three topographic variables, and one vegetation variable (Table S2).

Nineteen bioclimatic variables were downloaded from the World Climate Database (https://www.worldclim.org). The WorldClim 1.4 dataset (Hijmans et al., 2005) was selected for paleoclimatic data, including the LGM and the mid‐Holocene (MH; ~6000 years BP), the data were based on the Coupled Model Intercomparison Project Phase 5 (CMIP5). Since only CCSM4, MIROC–ESM and MPI–ESM–P Global Climate Models (GCMs) were available for the data of the LGM, to compare the prediction results of different GCMs, we selected the above three GCMs to simulate the ancient distribution of *Polyspora*. CCSM4 (The Community Climate System Model version 4) (Gent et al., 2011) is one of the most effective GCMs for predicting the impact of climate change on the distribution of animal and plant (Geng et al., 2022), and has the best precipitation prediction performance (Yang et al., 2020), especially for the precipitation prediction in southwest China (Yang, Yong, et al., 2021). MIROC–ESM (the Model for Interdisciplinary Research on Climate Earth System Model) has a good simulation of terrestrial carbon cycle and vegetation dynamics (Watanabe et al., 2011), and a good prediction of rainfall in the Yangtze River basin of China (Yang et al., 2020). MPI–ESM–P (Max Planck Institute for Meteorology Paleoclimate Model) is a model specially designed for paleoclimate simulation (Braconnot et al., 2012; Jungclaus et al., 2013), which is accurate in simulating the trend of extreme temperature change in China (Jiang et al., 2017).

WorldClim 2.1 dataset (Fick & Hijmans, 2017) was selected for current (the average between 1970 and 2000) and future (2081–2100) climate data. The data were based on the Coupled Model Intercomparison Project Phase 6 (CMIP6). The WorldClim 2.1 dataset was released in January 2020. Compared with CMIP5, the simulation results of CMIP6 are closer to the actual observation results (Fan et al., 2020; Gao et al., 2021), which improves the simulation capability of regional temperature and precipitation in China (Zhu et al., 2021). Four GCMs and four shared socio‐economic pathways (SSPs) were used for future climate data. The four GCMs were the Euro‐Mediterranean Center on Climate Change Earth System Model Version 2 (CMCC–ESM2) (Lovato et al., 2022), the Centre National de Recherches Meteorologiques Model Version 6.1 (CNRM–CM6‐1) (Brient et al., 2019), the European Community Earth‐system Model version 3.3 for vegetation (EC‐Earth3‐Veg) (Döscher et al., 2022) and the Higher‐Resolution Version of the Max Planck Institute Earth System Model version 1.2 (MPI–ESM1‐2–HR) (Müller et al., 2018). CMCC–ESM2 and MPI–ESM1‐2–HR are more accurate in predicting temperature in China, while EC‐Earth3‐Veg and CNRM–CM6‐1 are more accurate in predicting precipitation in China (Yang, Zhou, et al., 2021; Zhu et al., 2021). There are four shared socio‐economic pathways (SSPs) for future climate, including SSP126, SSP245, SSP370, and SSP585. SSPs can better reflect the correlation between socio‐economic development and climate scenarios (Gao et al., 2021). Under these pathways, global warming will be 3–5°C by 2100 (Hausfather, 2020). The SSP126 scenario is a sustainable green path with global warming of 3–3.5°C by 2100; the SSP245 scenario is middle road, the world follows a path in which social, economic, and technological trends do not shift markedly from historical patterns, with a warming range of 3.8–4.2°C by the end of the century; the SSP370 scenario is a regional competition route, with countries focusing on regional development, and a warming of 3.9–4.6°C by 2100; the SSP585 scenario envisages the world in which both economic output and energy consumption grow rapidly and without limit, with global warming of 4.7–5.1°C by 2100.

In total, six UV‐B radiation variables were obtained from the Global UV‐B radiation database (gIUV, https://www.ufz.de/gluv/) (Beckmann et al., 2014). Seven soil quality data were downloaded from the Harmonized World Soil Database v 1.2 (HWSD, https://www.fao.org/soils‐portal/data‐hub/soil‐maps‐and‐databases/harmonized‐world‐soil‐database‐v12/zh/) (Fischer et al., 2008). Among the three topographic variables, the elevation variable was obtained from the WorldClim v 2.1 dataset, the slope and aspect variables were extracted by the ArcGIS 10.2 (http://www.esrichina.com.cn/) spatial analysis function based on the elevation variable. Vegetation data were downloaded from Resources and Environmental Science and Data Center, Chinese Academy of Sciences (RESDC, http://www.resdc.cn).

Except for 19 bioclimatic variables, we assumed that other environmental variables remained constant in different periods, because the changes of these variables lag far behind climate change and data are lacking (Shabani et al., 2019; Wu et al., 2015, 2021).

The temperature value in the original layer of paleoclimatic variables were reduced by 10 times with the R package “terra” (Robert, 2022), which was unified with the current and future climate layers. All the environmental variables were trimmed to China region using R package “terra” (Robert, 2022), resamped to a unified spatial resolution of 2.5′, and converted to ASC format. China region was based on the Chinese vegetation layer obtained by RESDC. All the coordinate systems used in this study were the World Geodetic Coordinate System 1984 (WGS1984).

**FIGURE 3:**
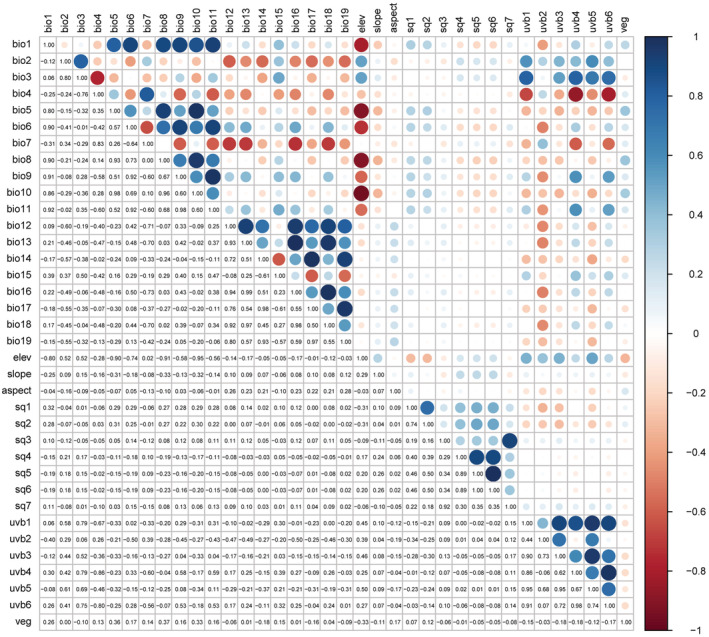
Correlation analysis of environmental variables for *Polyspora*. The lower left part is the correlation value *r*, ranging from −1 to 1, positive values indicate positive correlation, negative values indicate negative correlation, and 0 indicates non correlation. The greater the |*r*|, the stronger the correlation. The upper right part is a graphical transformation of correlation values. Positive correlations are displayed in blue and negative correlations in red color. Color intensity and the size of the circle are proportional to the correlation coefficients (see the right‐hand legend).

To avoid over‐fitting or inaccurate modeling of MaxEnt model due to strong correlation between environmental variables (Hu & Liu, 2014; Li et al., 2020), the correlation between climate factors were comprehensively tested by using R package “corrplot” (Wei & Simko, 2021) and the contribution rate of climate factors generated by MaxEnt v3.4.4 software. The selection principle of climate factors used in MaxEnt model calculation were: (1) the climate factors with contribution rate >10% were retained; (2) between pairs of strongly correlated environmental variables (|*r*| ≥ 0.8), the variables with large contribution rate and biological significance were retained. After screening, 17 environmental factors (seven climatic factors, two UV‐B radiation factors, four soil factors, three topographic factors, and one vegetation factor) were obtained for genus level prediction of *Polyspora* (Figure 3, Table S3); 14–19 environmental factors were selected for species‐level prediction (Table S3, Figures S1–S5).

### 2.3 MaxEnt species distribution modeling

**FIGURE 4:**
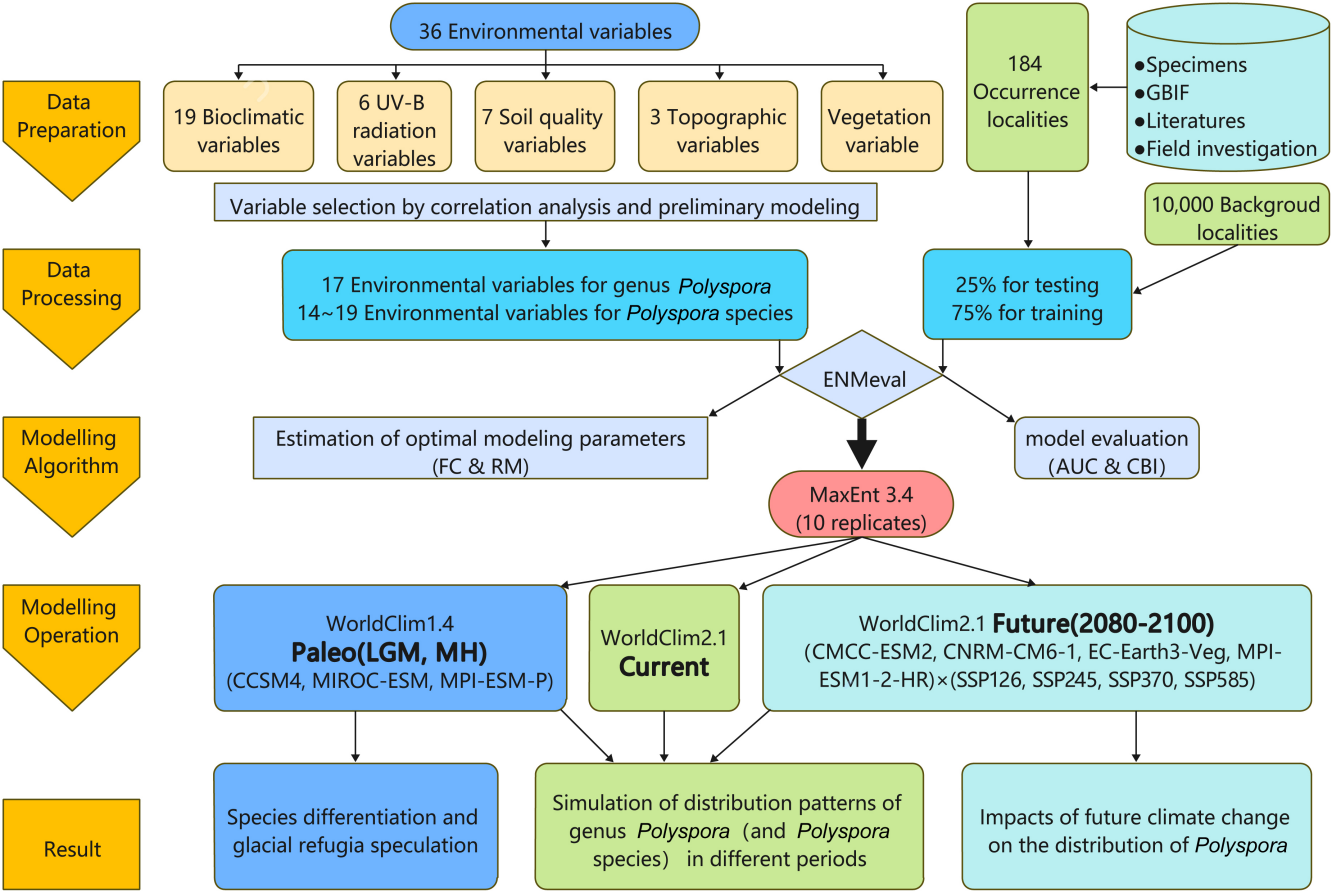
Working flow chart of niche simulation.

The genus *Polyspora* in China mainly grows in tropical and subtropical forests. Different species have different distribution habitats and altitudes. For example, *P. longicarpa* is mainly distributed in the mid‐montane humid ever‐green broad‐leaved forest above 2000 m altitude, while *P. hainanensis* is distributed in the tropical rainforests around 600 m altitude. There are different degrees of interspecific differences in climate, UV‐B, soil, topography, etc., the distribution range and quantity of each species are different. According to the different habitat requirements of species, to accurately simulate the suitable habitat of this genus and each species in different periods, we established the workflow chart and ODMAP of niche simulation (Figure 4, Appendix A) based on the standard protocol of species distribution model (Zurell et al., 2020). The entire workflow of data source and preprocessing, modeling parameter generation, model calculation, and performance verification were described in detail.

Geographical distribution data of *Polyspora* species and the climate data of four periods were imported into MaxEnt v3.4.4, 75% of the distribution points were randomly selected for modeling, and the remaining 25% were used for verification. The maximum number of iterations was set to 1000, background points 10,000, replications bootstrap 10, and use the average value of 10 operations to ensure the reliability of the results. The R package “ENMeval 2.0” (Kass et al., 2021) was used to optimize two key modeling parameters: regularization multiplier (RM) and feature combination (FC). For *Polyspora* and *P. axillaris*, RM was set to 0.5–4, with an interval of 0.5 each time, a total of 8 values. The RM of *P. chrysandra*, *P. Hainanensis*, *P. longicarpa*, and *P. speciosa* were set to 1–4 with each interval of 0.5, a total of 7 values. RM = 0.5 had been tested for these species, but the results were not desirable, and the difference between the running results of 0.5 and 1 was too large. We selected six types for FC testing, which were L, LQ, LQH, H, LQHP, and LQHPT. Among them, L means linear, Q means quadratic, H means hinge, P means product, and T means threshold. Therefore, a total of 42 to 48 sets of parameter combinations were used for the ENMeval test. For *Polyspora*, *P. axillaris*, *P. chrysandra*, and *P. speciosa*, the “block” partition method was used to perform ENMeval operation to limit the autocorrelation in the large scale space. Since the sample sizes of *P. hainanensis* and *P. longicarpa* were <25, the “Jackknife” method was used for ENMeval operation to generate the maximum available information model and improve the prediction accuracy (Galante et al., 2018; Pearson et al., 2007; Shcheglovitova & Anderson, 2013). The model performance was evaluated according to akaike information criterion (AICc), delta akaike information criterion (delta AICc), the difference between training areas under curves (AUCs) and testing AUC (AUC.diff), and the 10% training commission rate (OR10). The model with the lowest AICc (delta.AICC = 0) and the higher AUC value (AUC > 0.9) was considered to be the best model. Since models with delta.AICc < 2 are all reliable (Phillips et al., 2017), to improve the reliability of models, we also considered models with smaller AUC. Diff and OR10 for some species (Warren & Seifert, 2011).

The simulation accuracy was evaluated by the value of the area under the receiver operating characteristic curve (AUC) (Lobo et al., 2008) and the continuous Boyce index (CBI) (Boyce et al., 2002). The range of AUC values is [0, 1], and the closer it is to 1, the more accurate the simulation results are (Jiang et al., 2016). AUC > 0.9 indicates that the simulation result is very accurate, and the simulation results with AUC < 0.7 are not credible (Elith et al., 2006). The range of CBI value is [−1, 1], where positive values indicate an accurate model, zero indicates no difference from random, and negative values indicate a negative correlation (Lake et al., 2020). To ensure better model performance, we selected the model with AUC > 0.9 and CBI > 0.5.

Simulation results of MaxEnt were imported into ArcGIS, and the ASCII format was converted into raster layer by conversion tools, and the suitable area was divided by reclassify tools. According to the calculation results of MaxEnt model, the suitability grades of *Polyspora* were divided into four regions: unsuitable region (0–0.1), lowly suitable region (0.1–0.3), moderately suitable region (0.3–0.5), and highly suitable region (0.5–1) (Sun et al., 2020; Wu et al., 2021; Zhao et al., 2020).

In the simulation of centroid transfer, suitable area change and interspecific overlapping distribution area, the commonly used fixed threshold of 0.5 was used as the critical value of species distribution/non distribution (Jiménez‐Valverde & Lobo, 2007). Considering that the selection of GCMs would lead to the uncertainty of the prediction results, we performed arithmetic average processing on the prediction results of three paleo GCMs and four future GCMs, and superimposed the prediction results of different models under the same climate scenario into a single graph. We used the Centroid Changes tool in SDMtoolbox V2.4 Toolkit (Brown et al., 2017) to analyze the changes of distribution centroid of *Polyspora* in different periods and different SSPs, and used Distribution Changes Between Binary SDMs to analyze the changes of suitable areas of *Polyspora* in different periods.

In the process of species differentiation and glacial refugia speculation of *Polyspora*, the ASCII files were converted into binary SDM layers, and the suitable habitats of different species (threshold exceeds 0.1) were reclassified. Then, the overlapping and independent distribution regions of different species in different periods were obtained by the superposition statistics of pixel metadata. The regions with the largest number of overlapping species during the glacial period were the possible areas of species differentiation. SDMtoolbox was used to analyze the changes of suitable areas during the LGM and current period for *Polyspora* species, and inferred their possible glacial refugia for each species.

### 2.4 Phylogenetic analysis and differentiation time estimation

To better reveal the phylogeny and species differentiation relationship of Chinese *Polyspora*, and confirm the simulation results of MaxEnt model, the complete chloroplast genomes of six *Polyspora* species were downloaded from GenBank, of which two species (*P. tiantangensis* and *P. chrysandra*) were assembled, annotated and uploaded by our team, the chloroplast genome sequence of *Apterosperma oblata* was also downloaded for used as an outgroup in the phylogenetic tree (Table S4). We aligned the seven complete chloroplast genomes with MAFFT v7.450 (Katoh & Standley, 2013), selected the best nucleotide substitution model (GTR + I + G) with modelgenerator v0.85 (Keane et al., 2006), then constructed the Maximum Likelihood (ML) tree with 1000 bootstrap replicates by RAxML v8.2.12 (Stamatakis, 2014) on CIPRES Science Gateway platforms (Miller et al., 2010). Species differentiation time was calculated in Beast v2.6.6 (Bouckaert et al., 2019) using a loose molecular clock. The parameter file (.xml) were constructed with BEAUti 2. The main parameters were set as follows: Nucleotide substitution model was GTR; Clock. rate was 1.52e‐8; Tree prior was Birth‐Death model; Calibration point of differentiation time: according to the research results of Rose et al. (2018), the differentiation time of *Polyspora* and *Apterosperma* was set as 23.8 Ma (Rose et al., 2018); according to the research results of Yu et al. (2017), the earliest differentiation time of Chinese *Polyspora* was set as 8.33 Ma (Yu et al., 2017), and used the 95% HPD as a range; MCMC: running total algebraic 10,000,000 generations, sampling frequency (Log parameters every) 10,000 times, the log every in tracelog/screenlog/treelog were set to 10,000 times. Tracer v1.7.2 (Rambaut et al., 2018) was used to check whether the parameters converged and ensured that the ESS values of all parameters were >200. We used TreeAnnotator v2.6.6 to obtain tree, the burning percentage was set to 10%, MCC tree was selected for the target tree type, and median height was selected for the node height. Finally, we used FigTree v1.4.4 (http://tree.bio.ed.ac.uk/) to view and beautify the tree.

## 3 RESULTS

### 3.1 Optimal model and model accuracy evaluation

**FIGURE 5:**
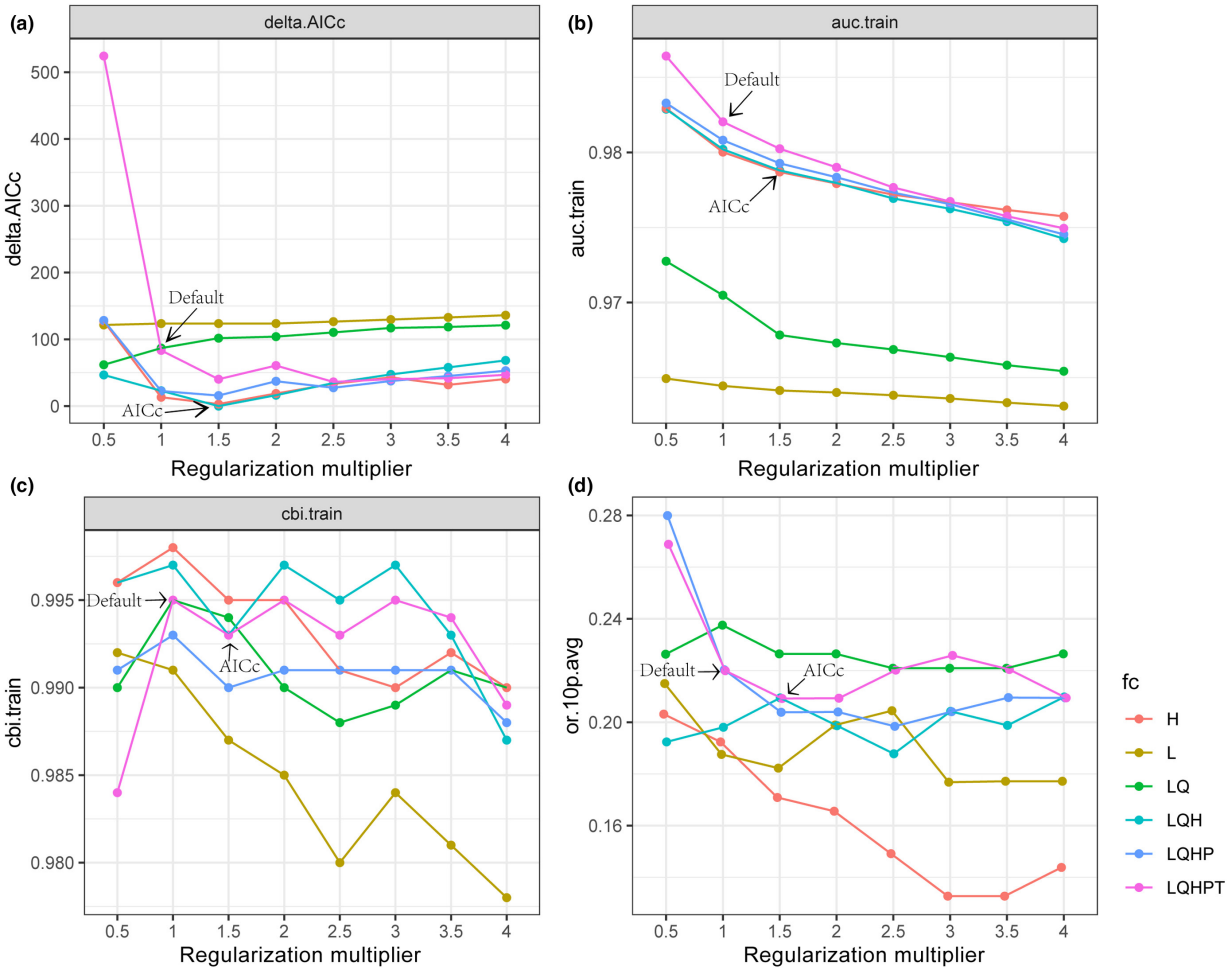
Optimization results for MaxEnt mode of *Polyspora* under different parameter settings, (a) delta.AICc, (b) AUC.train, (c) CBI.train and (d) 10% omission rate, or 10p.avg. Feature classes (H, hinge; L, linear; LQ, linear quadratic; LQH, linear quadratic hinge; LQHP, linear quadratic hinge product; LQHPT, linear quadratic hinge product threshold).

Under the default parameter (RM = 1 and FC = LQHPT) of MaxEnt, delta AICc = 83.400, AUC = 0.982, CBI = 0.995, AUC.diff = 0.120, OR = 0.219. When RM = 1.5 and FC = LQH, delta AICc = 0, AUC = 0.979, CBI = 0.993, AUC.diff and OR10 were lower than the default parameters (Figure 5, Table S5). The optimized parameters significantly reduced the model complexity and were more suitable for modeling migration in different periods. Therefore, RM = 1.5 and FC = LQH were selected as modeling parameters for niche simulation of *Polyspora* in this study. Similar methods were used to select the optimal modeling parameters for niche simulation of each species in the genus *Polyspora*. The results were shown in Tables S3 and S6–S10, and Figures S6–S10.

The selected parameter combinations were used to simulate and predict the historical, contemporary, and future distribution areas of *Polyspora* species. The AUC values of the training set and test set in each period, each GCM and each SSP were all >0.9, and the CBI values were all >0.5, indicating that the model had good‐fitting effect and high‐prediction accuracy.

### 3.2 Contribution of environmental variables to species distributions

**FIGURE 6:**
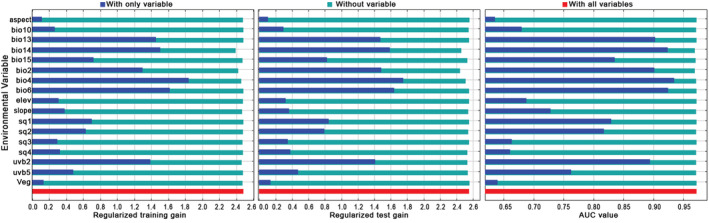
Results of the Jackknife test of environmental variables' contribution in *Polyspora*'s potential geographical distribution. The figure displays the result of the Jackknife test of variable contribution using regularized training gain, regularized test gain, and AUC value, respectively. The blue bars indicate the gain using the sole environmental variable, the green bars indicate the gain excluding the single variable, and the red bars indicate the gain including all variables.

Figure 6 shows the results of Jackknife test. When a single variable was used, those with the largest regularization training gain, regularization test gain, and AUC value were temperature seasonality (Bio4), min temperature of coldest month (Bio6), and precipitation of driest month (Bio14).

**FIGURE 7:**
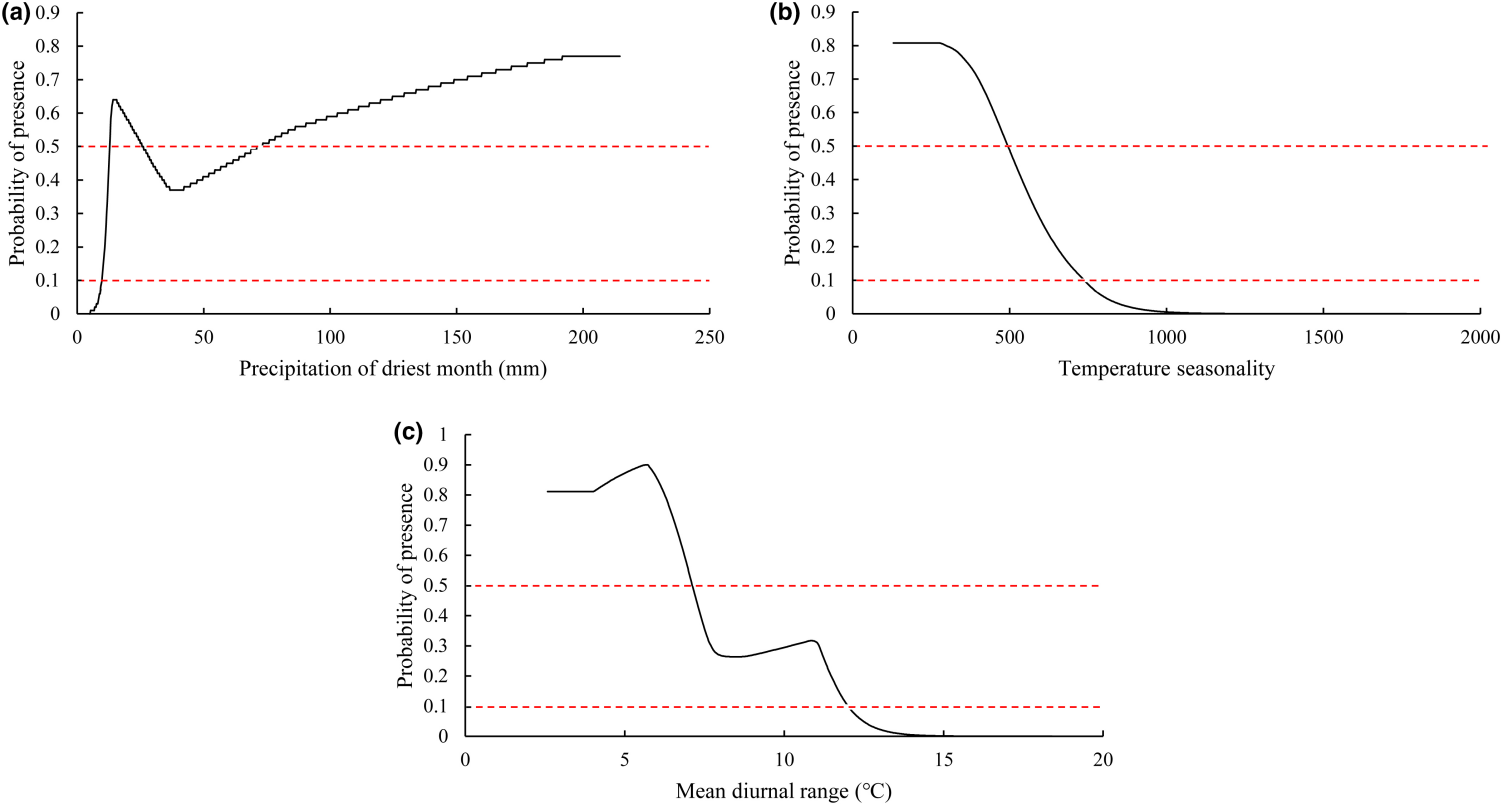
Response curve of the dominant environmental variables. (a) Precipitation of driest month, (b) temperature seasonality, and (c) mean diurnal range. The red dashed line at the top of each mini‐graph indicates the highly suitable distribution range line with a threshold of 0.5, and the red dashed line at the bottom represents the suitable distribution range line with a threshold of 0.1.

The percentage contribution rate of environment variables to MaxEnt modeling were obtained from the model output (Table S11). The top three percentage contribution rates of single environmental variables were Bio14 (48.3%), Bio4 (36.5%), and Bio2 (3.6%), with a cumulative contribution rate of 88.4%. Among the environmental factors applied, the contribution rate of temperature factors were 41.1%, the contribution rate of precipitation factors were 49.9%, and the total contribution rate of climate factors were 91%. According to the response curve of environmental factors to the presence probability in MaxEnt model (Figure 7), taking the presence probability of 0.5 as the threshold value of the highly suitable area, *Polyspora* was most likely to be found when precipitation of driest month was between 13 and 25 mm, and when temperature seasonality was <493, and when mean diurnal range <7°C.

Bioclimatic factors also played a dominant role in the distribution prediction of Chinese *Polyspora* species, with an average cumulative contribution rate of 84.5% for the five species. Except for *P. axillaris*, the influence of temperature was greater than that of precipitation. Especially in *P. hainanensis*, the cumulative contribution rate of temperature factors were up to 91.5%. In the case of *P. axillaris*, precipitation had a greater impact. In addition to climate factors, UV‐B also had a greater impact on *P. axillaris*, with a cumulative contribution rate of 18%. Topographic factors had a great impact on *P. speciosa* and *P. longicarpa*, with cumulative contribution rates of 12.1% and 11.7%, respectively (Table S11).

The environmental factors that play a dominant role in the distribution of *P. axillaris* were Bio12 (43.7%), Bio2 (19.9%), Bio7 (12.3%), UVB4 (12%), and UVB2 (5.6%), with a cumulative contribution rate of 93.5%. *Polyspora axillaris* could only survive in areas with an annual rainfall of more than 1478 mm, while the annual rainfall in high‐suitable areas was more than 2000 mm. The mean UV‐B of lowest month in the highly suitable area of *P. axillaris* was above 2346 J·m^−2^·day^−1^, and the UV‐B seasonality should not exceed 102,738 J·m^−2^·day^−1^. The key environmental factor affecting the distribution of *P. chrysandra* was Bio3 (65.1%), and the Isothermality in high‐suitable area should be >49. The main environmental factors affecting the distribution of *P. speciosa* were Bio2 (35.8%), Bio14 (27.6%), Bio4 (14.5%), and elevation (11.1%). The range of environmental variables in high‐suitable areas were: Bio2, <7°C; Bio14, 16–37 mm; Bio4, 617–755; elevation, 476–1212 m. The top three factors affecting the distribution of *P. hainanensis* were all temperature factors, which were Bio7 (61.1%), Bio6 (25.3%), and Bio4 (5.1%), respectively. The temperature range of the high‐suitable area of *P. hainanensis* were: temperature annual range below 16°C, min temperature of the coldest month above 14°C, temperature seasonality below 338. The environmental factors that play a major role in the distribution of *P. longicarpa* were Bio3 (29.6%), Bio4 (24%), Bio6 (11.4%), Bio17 (10%), UVB4 (7%), and elevation (4.6%). The range of environmental variables in the high‐suitable area of *P. longicarpa* were: isothermality more than 46, temperature seasonality range from 341 to 464, min temperature of the coldest month range from −3 to 3°C, precipitation of the driest quarter range from 47 to 106 mm, mean UV‐B of lowest month was below 2479 J·m^−2^·day^−1^.

### 3.3 Simulation of distribution patterns of genus *Polyspora* in different periods

#### 3.3.1 Potential suitable habitat of *Polyspora* under current climate scenarios

**FIGURE 8:**
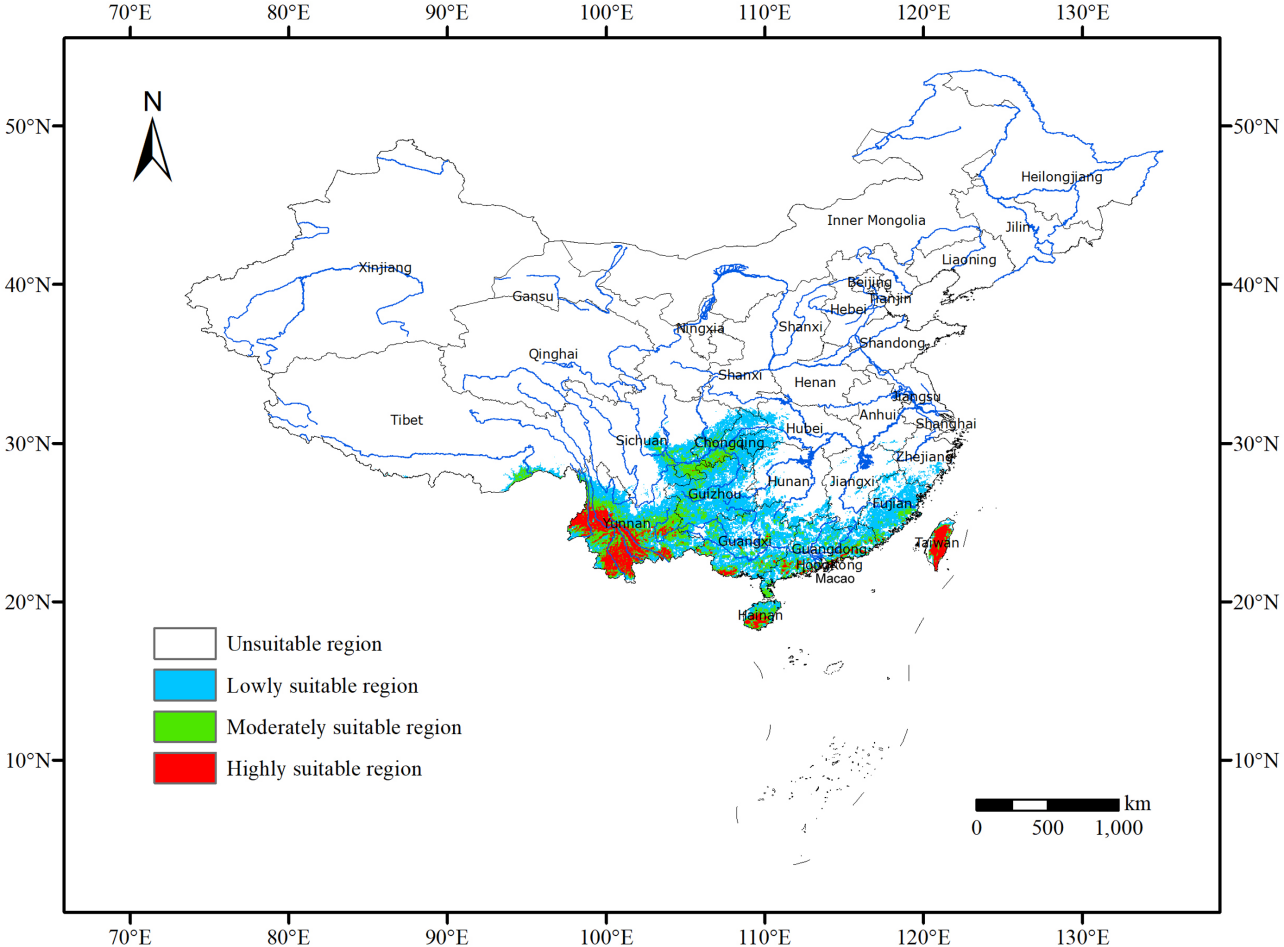
Potential distribution areas of Chinese *Polyspora* under current environmental conditions. When presence probability is <0.1, unsuitable region; when presence probability is 0.1–0.3, lowly suitable region; when presence probability is 0.3–0.5, moderately suitable region; and when presence probability is >0.5, highly suitable region.

At present, *Polyspora* was mainly distributed in southwest China, south China, and Taiwan Province (Figure 8). The highly suitable area was mainly distributed in the southwestern Yunnan, Hainan Island and Taiwan Island, with an area of 128,014 km^2^, accounting for 1.3% of China's total land area. The moderately suitable area was mainly located in the Sichuan Province, Chongqing Municipality, Fujian Province, Guangdong Province, Guangxi Zhuang Autonomous Region and southeastern Tibet, with an area of 240,357 km^2^, accounting for 2.5% of China's land area. The low‐suitability area was located at the periphery of the moderately suitable area, with an area of 705,259 km^2^ (Table S12).

#### 3.3.2 Potential suitable habitat of *Polyspora* under paleoclimate scenarios

**FIGURE 9:**
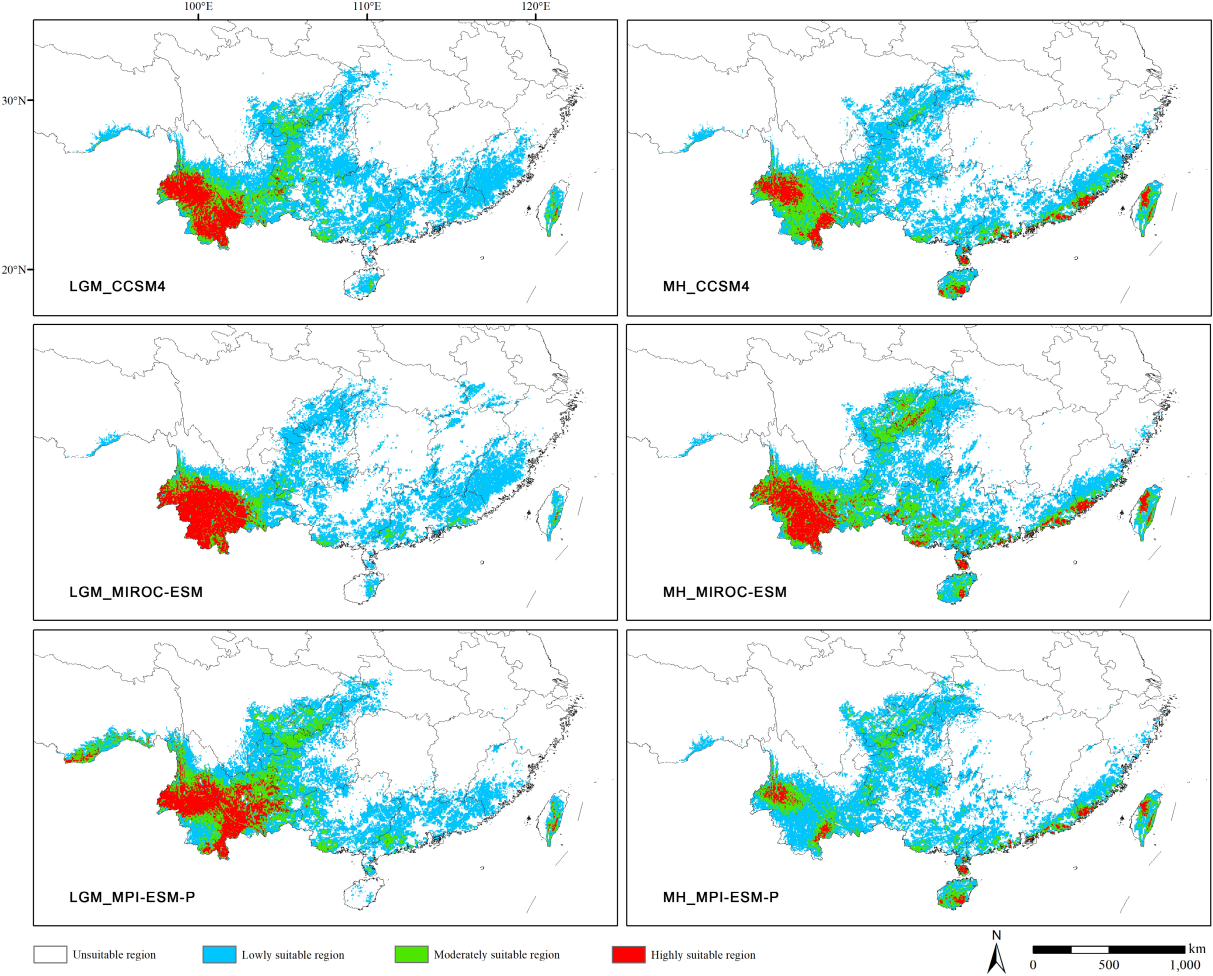
Potential distribution areas of Chinese *Polyspora* under paleoclimate scenarios. Comparison of three global climate models (CCSM4, MIROC–ESM, and MPI–ESM‐P) in two paleoclimatic periods, the last glacial maximum and mid‐Holocene.

The distribution simulation of *Polyspora* during LGM period using three GCMs showed that the highly suitable habitats were mainly located in Yunnan Province. In CCSM4 and MIROC–ESM, the highly suitable habitat was mainly located in southwestern Yunnan. While in MPI–ESM‐P, the highly suitable area was significantly shifted to the eastern Yunnan (Figure 9).

**FIGURE 10:**
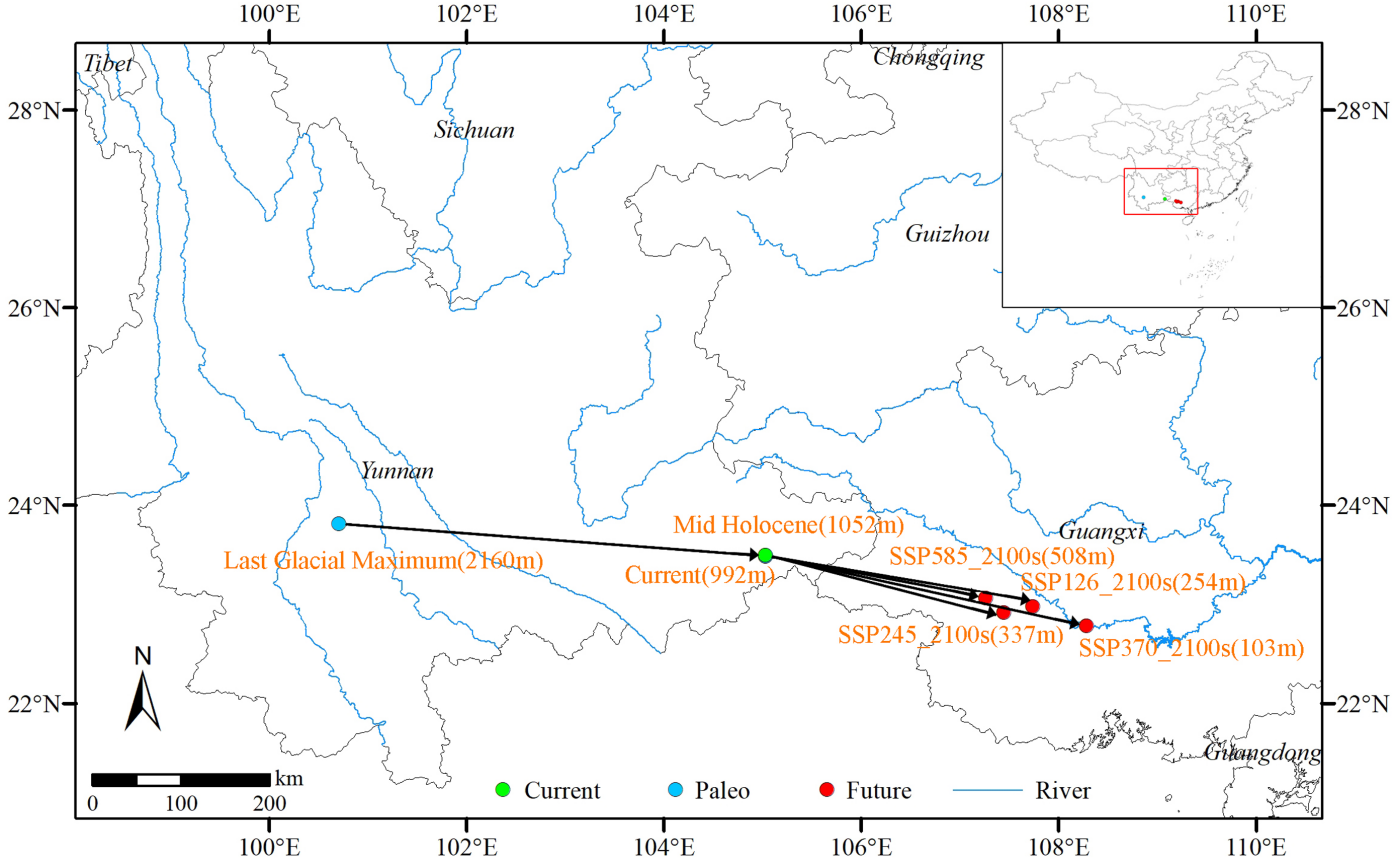
Shifts of centroids of *Polyspora* under different periods and scenarios. The black arrow lines in the map are the centroid transfer routes, and the arrows indicate the transfer directions. Brown fonts indicate the period names, and the future period includes four SSPs, marked before 2100s. The numbers in parentheses indicate the elevation of the centroid locations.

In the MH, the habitats of *Polyspora* under the three GCMs were close to the current suitable habitats, with almost overlapping centroids (Figure 10), but the suitable habitat area was less than current. Especially in MPI–ESM‐P, the highly suitable habitat area was only 29,854 km^2^ (Figure 9, Table S12). Compared with the LGM, the highly suitable distribution area expanded eastward, and the distribution centroid also shifted eastward (Figure 10).

#### 3.3.3 Potential suitable habitat of *Polyspora* under future climate change scenarios

In the future, with the increase of greenhouse gas emissions, except for EC–Earth3‐Veg_SSP585 scenario, the total suitable area of *Polyspora* in other GCMs and SSPs decreased to varying degrees, while the highly suitable area decreased or expanded due to different GCMs and SSPs.

**FIGURE 11:**
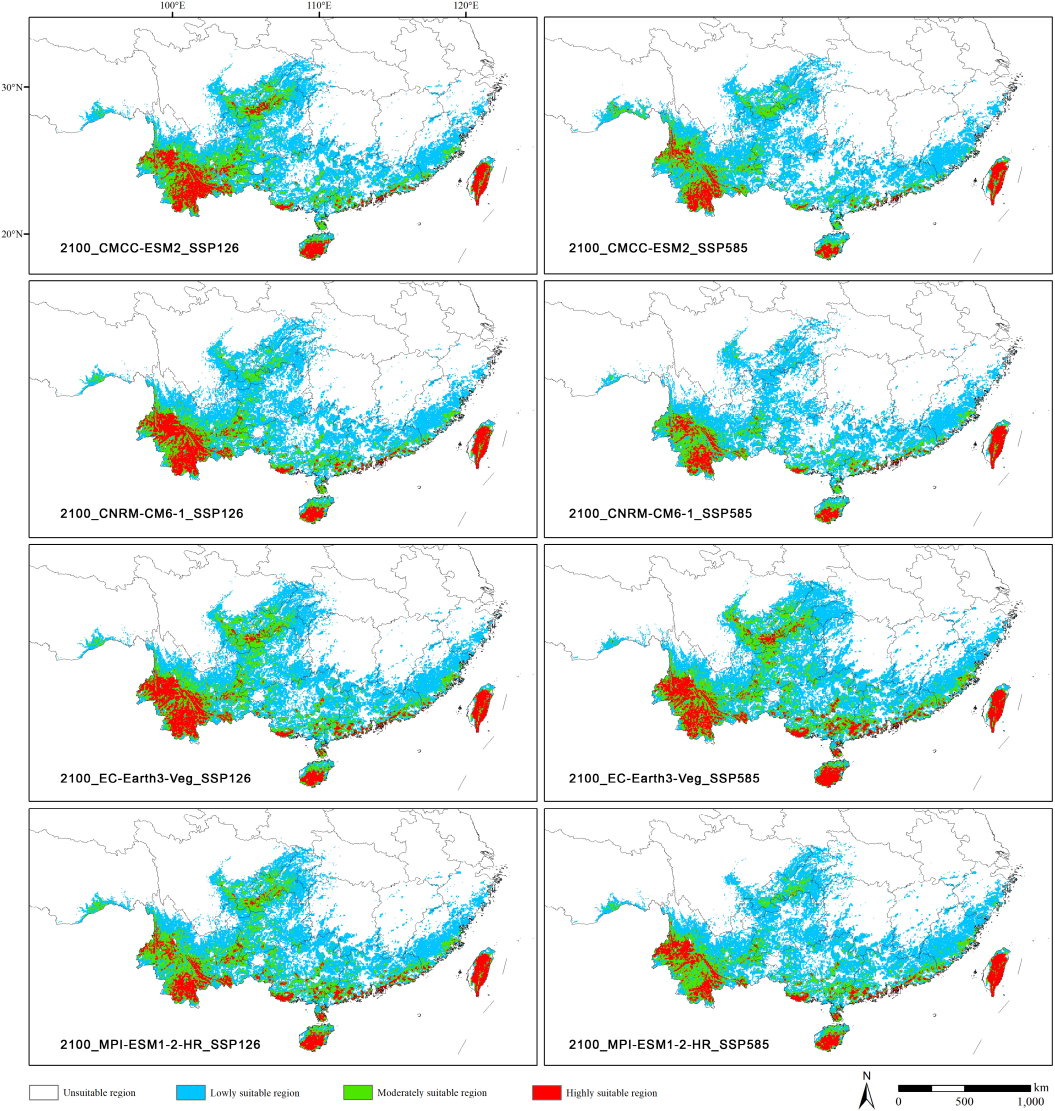
Potential distribution areas of Chinese *Polyspora* under future climate scenarios. Future projections (2100) are estimated from four global climate models (CMCCESM2, CNRM–CM6‐1, EC‐Earth3‐veg and MPI–ESM1‐2–HR) and two shared socio‐economic pathways (SSP126 and SSP585). Left panels represent the most optimistic path (SSP126) and right panels represent the most pessimistic path (SSP585).

Under the SSP126 scenario, the total suitable area predicted by different GCMs decreased. Except for the MPI–ESM1‐2–HR model, the highly suitable area predicted by the other three GCMs increased by 2.78% (CMCC–ESM2) to 15.97% (EC–Earth3‐Veg) compared with the current scenario (Figure 11, Table S12).

Under the SSP585 scenario, except for the EC–Earth3‐Veg, the highly suitable area predicted by the other three GCMs would be lost to varying degrees, with loss rates ranging from 12.95% (MPI–ESM1‐2–HR) to 36.71% (CNRM–CM6‐1) (Figure 11, Table S12). However, the EC–Earth3–Veg model predicted that the highly suitable area would increase by 26.83%.

Under two intermediate scenarios (SSP245 and SSP370), the highly suitable area predicted by most GCMs also shown a decreasing trend (Table S12).

**FIGURE 12:**
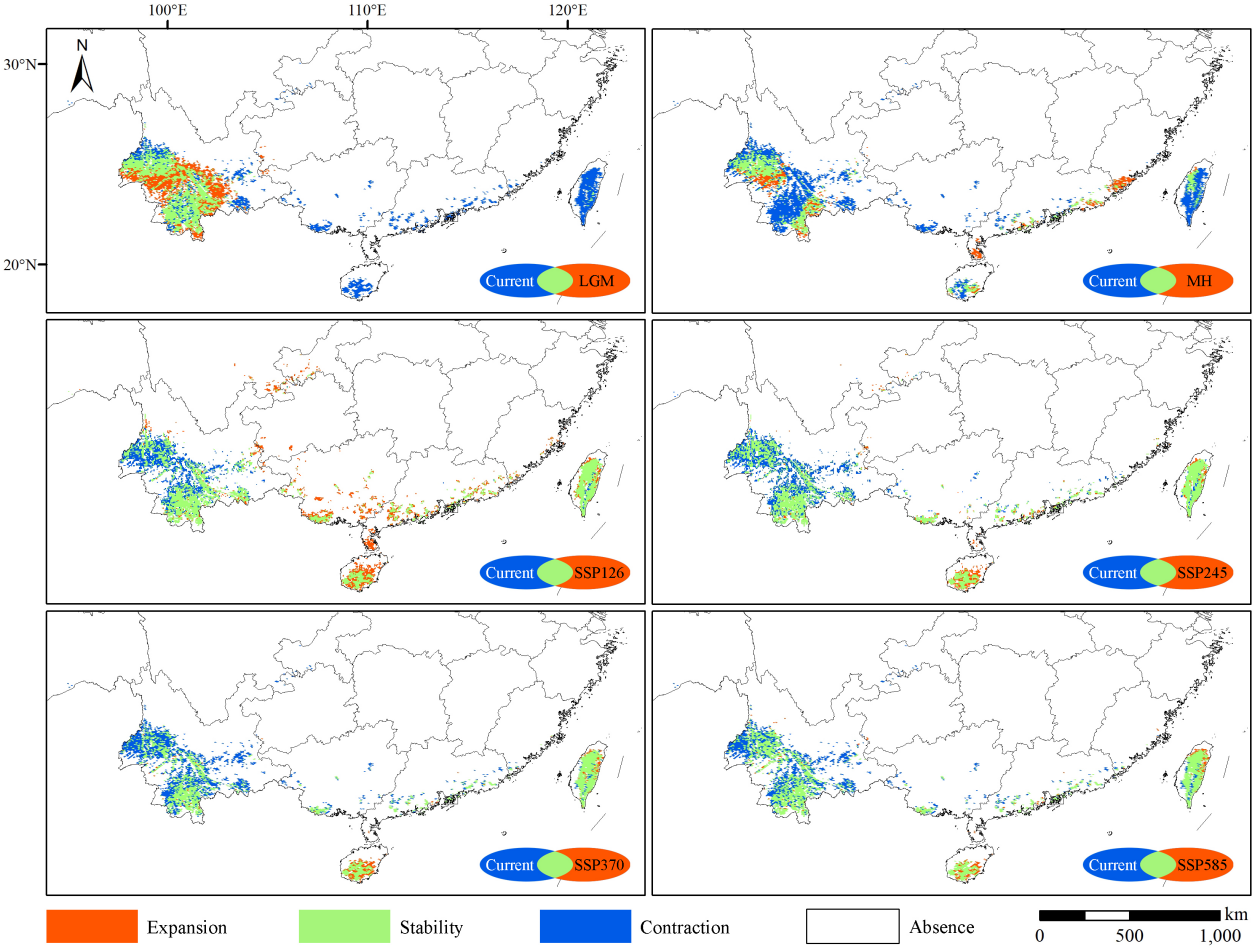
Niche overlap map of *Polyspora* between current and other periods. Simulated distribution maps of paleo and future climate periods are the arithmetic average superimposed maps of different global climate models. Green color on the map indicates stable habitat of *Polyspora* in each period and SSP compared with the current. Blue color indicates contraction habitat of *Polyspora* in each period and SSP compared with the current. Orange indicates expansion habitat of *Polyspora* in each period and SSP compared with the current.

In the future, under different SSPs, the habitat stability of the genus *Polyspora* in Taiwan and southern Yunnan would be relatively high, the highly suitable area in the western Yunnan would contract, and the highly suitable area in Chongqing, Lingnan and southeast China would expand to varying degrees, Hainan Island would expand most obviously, and the highly suitable area would generally move to the southeast (Figures 11 and 12). It could be seen from the centroid distribution map that under different SSPs in the future, the distribution centroids of *Polyspora* would all move southeast to Guangxi. The location of the centroids under different SSPs were very close (Figure 10).

### 3.4 Simulation of distribution patterns of *Polyspora* species in different periods

**FIGURE 13:**
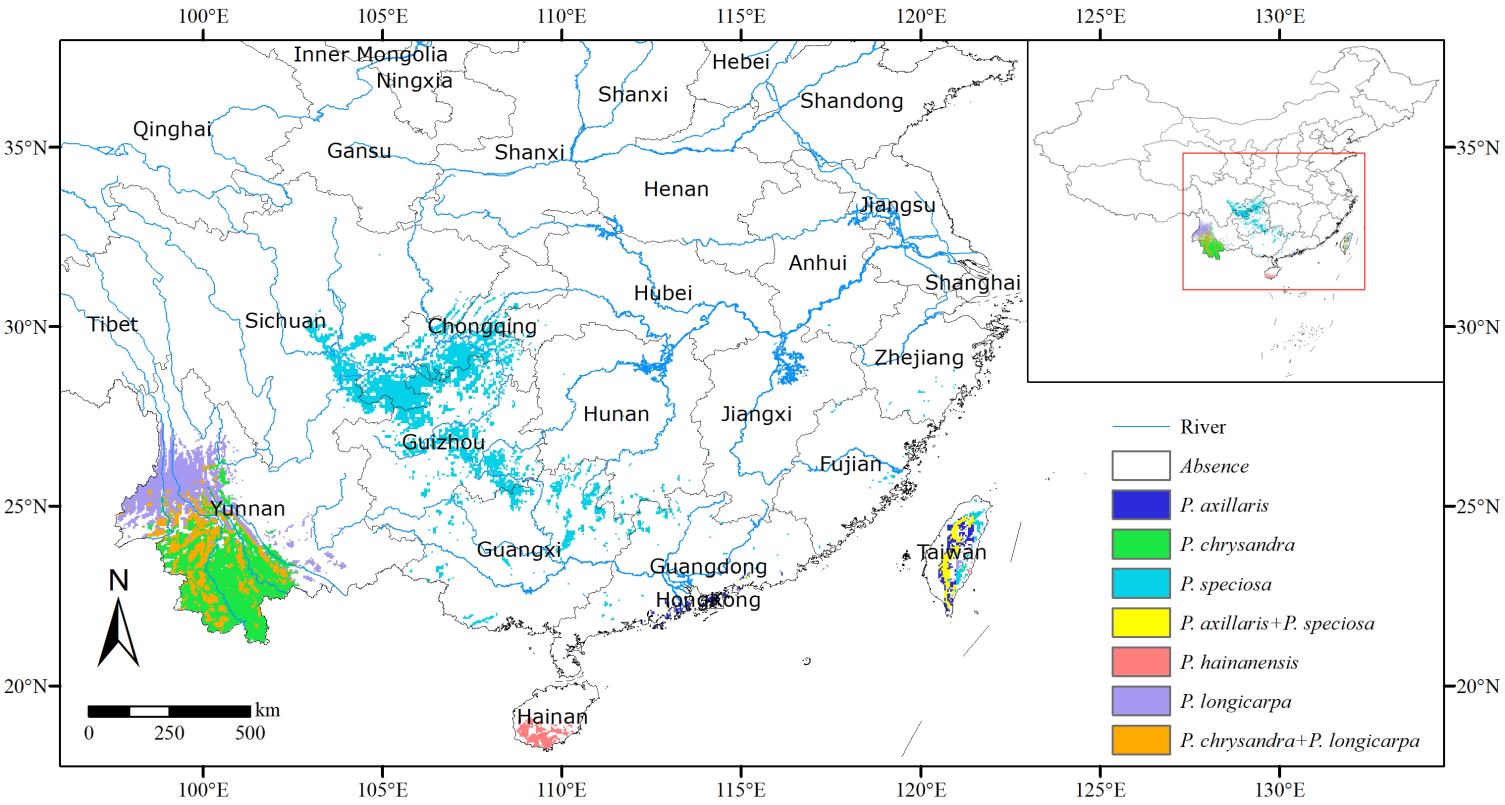
Potential highly suitable habitat of *Polyspora* species in China. Different colors represent highly suitable habitats for different species, among which yellow and orange indicate highly suitable habitats shared by two species.

The current potential highly suitable areas of five species of *Polyspora* (*P. axillaris*, *P. chrysandra*, *P. speciosa*, *P. hainanensis*, and *P. longicarpa*) were species‐specific (Figure 13, Figure S11). Highly suitable areas of *P. axillaris* were located in southeastern Guangdong and Taiwan. The highly suitable area of *P. hainanensis* was only located in the Hainan Island. Highly suitable areas of *P. chrysandra* and *P. longicarpa* were both located in the west and southwest Yunnan, and the two species have a large area of overlapping distribution. *Polyspora speciosa* had the widest range of high suitability, which were located in southeast Sichuan, Chongqing, Guizhou, northern Guangxi and Taiwan. *Polyspora axillaris* and *P. speciosa* overlapped slightly in Taiwan.

Since the LGM, *P. axillaris* had experienced a large expansion, and the optimal distribution area in the MH was slightly higher than that in the current period (Figure S12). At present, highly suitable habitat for *P. axillaris* was located in the Pearl River Delta and Taiwan (Figure S13). In the future, under different SSPs, the total suitable area and highly suitable area would both increase significantly, and expand eastward and westward at the same time. Under EC–Earth3–Veg_SSP585 scenario, the highly suitable area would reach 110,746 km^2^ in 2100 (Figures S14 and S15, Table S13).

Under the three GCMs of paleoclimate, the potential suitable areas of *P. chrysandra* during the LGM were more than that of current period (Figure S16, Table S14). From LGM to MH, the suitable habitat decreased, and then expanded after the MH. Currently, highly suitable areas were located in southwestern and southern Yunnan (Figure S17). In the future, except for the CMCC–ESM2_SSP245 scenario, the potential suitable habitat of *P. chrysandra* would continue to expand, with an average increase of 40,000 km^2^ by 2100, but the average loss of highly suitable habitat was 10,000 km^2^ (Table S14, Figures S18 and S19).

Highly suitable areas of *P. speciosa* during the LGM were mainly located in southeast Sichuan, Chongqing and northern Guizhou, and expanded to Guangxi in the MH period (Figure S20). At present, in addition to the above areas, there were also highly suitable habitats for *P. speciosa* in central and eastern Taiwan, and a large range of suitable habitats in eastern and southern China (Figure S21). In the future, under different SSPs, the total suitable area would continue to expand, but the highly suitable area would be shrunken in a small range under the CMCC–ESM2 and CNRM–CM6‐1 modes (Figures S22–S25, Table S15).

Current suitable habitats of *P. hainanensis* were mainly located in southern Hainan and eastern Taiwan, and the total suitable areas of *P. hainanensis* were less than other Chinese *Polyspora* species, which were 37,158 km^2^ (Figure S26, Table S16). The simulation results of three GCMs of paleoclimate showed that during the LGM, there was no moderately and highly suitable habitat for *P. hainanensis*, only lowly suitable habitat was found in southern Yunnan (Figure S27). In the MH, highly suitable habitat of *P. hainanensis* appeared in southern Hainan, while the suitable habitat in Yunnan was lost. With the future climate warming, the total suitable area of *P. hainanensis* would be reduced, but the highly suitable area would be relatively stable (Table S16, Figures S28 and S29).

During the LGM, highly suitable habitats of *P. longicarpa* were located in most areas of Yunnan and southern Taiwan, and under the LGM_MPI–ESM‐P model, there were also highly suitable habitats in southeastern Tibet (Figure S30). In the MH, the total suitable area decreased, but the highly suitable areas in the two paleoclimatic periods were more than that in the present period (Figure S30, Table S17). At present, *P. longicarpa* was mainly distributed in the western Yunnan and central Taiwan, with highly suitable area of 72,858 km^2^ (Table S17, Figure S31). Under various GCMs and SSPs combination in the future, except for the MPI–ESM1‐2‐HR_SSP126 model, the total and highly suitable areas of *P. longicarpa* would shrink westward by 2100. Under the CNRM–CM6‐1_SSP585 model, only 18,536 km^2^ would remain in the highly suitable area, with a loss rate of 74.56% (Figures S32 and S33, Table S17).

### 3.5 Phylogenetic analysis of the Chinese *Polyspora* based on the chloroplast genomes

**FIGURE 14:**
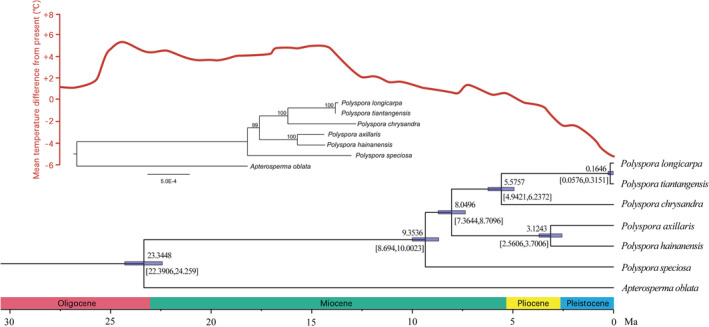
Phylogenetic relationships and divergent time estimated of Chinese *Polyspora* inferred from maximum likelihood analysis based on chloroplast genomes. The upper part shows the mean temperature difference between ancient and present. The middle part is the phylogenetic tree based on the maximum likelihood method, the numbers on the nodes indicate bootstrap values. The lower part is the divergent time of Chinese *Polyspora*, dark blue bars represent the node ages of 95% confidence interval.

The phylogenetic tree based on chloroplast genomes showed that the earliest branch of Chinese *Polyspora* was *P. speciosa*, and then it diverged into two big branches. Divergence time by Beast showed that the divergence between *P. axillaris* and *P. hainanensis* occurred at 3.12 Ma. At 5.58 Ma, *P. chrysandra* differentiated with *P. longicarpa* and *P. tiantangensis*. The differentiation of *P. longicarpa* and *P. tiantangensis* was the latest, and the divergence occurred during the Last Interglacial (LIG), 164 ka bp (Figure 14).

## 4 DISCUSSION

### 4.1 Key environmental factors shaping species distribution

Factors affecting species distribution include environmental factors, biological factors, and the characteristics of species themselves (Liu et al., 2013). Environmental factors mainly affect species distribution at large spatial scales (King et al., 2020). Biological factors and the characteristics of species themselves mainly affect the distribution of species in a relatively small scale (Zhu et al., 2013). The results of this study showed that *Polyspora* were widely distributed in the south of the Yangtze River, and the highly suitable area was mainly located in the south of 25° north latitude (Figure 8, current). The climate type of this area is mainly subtropical monsoon humid climate, and the vegetation type is mainly subtropical evergreen broad‐leaved forest, Theaceae is one of the typical representative plant group. Contemporary optimal distribution areas of *Polyspora* based on MaxEnt–ArcGIS were basically consistent with the regions with the highest species richness of Theaceae (Zhang et al., 2016). The main environmental factors affecting the distribution of *Polyspora* species were bioclimatic factors. Simulation results of MaxEnt showed that precipitation of driest month, temperature seasonality, and mean diurnal range affected the distribution of *Polyspora*. At the species level, except for *P. axillaris*, the influence of temperature on species distribution was greater than that of precipitation. The order of influence of various environmental factors was temperature > precipitation > UV‐B > topography > soil > vegetation. The dominant environmental factors of different species were different, resulting in different ecological niches occupied by each species, and then formed relatively independent distribution patterns. The south of the Yangtze River Basin in China has abundant rainfall, rain, and heat over the same period. The rainfall in the hottest season is large and concentrated (Liu et al., 2021), which is suitable for the growth of *Polyspora* species. The environmental factor that plays a leading role in the distribution of *P. axillaris* was annual precipitation, the annual precipitation in the Pearl River Delta region was 1600–2300 mm, and the annual precipitation in Taiwan Island was over 2500 mm, which created the best conditions for the growth of *P. axillaris*. UV‐B radiation has a significant impact on aboveground organs of plants, thereby limiting the distribution of species (Wu et al., 2021). In addition to bioclimatic factors, UV‐B radiation also played an important role in the distribution of *P. axillaris*. Isothermality was the primary ecological factor determining the distribution of *P. chrysandra* and *P. longicarpa*, these two species were mainly distributed in southern and western Yunnan, where the annual temperature difference was small and the monthly temperature varied greatly, providing a high isothermality for the species distribution. In addition to bioclimatic factors, elevation had a great impact on the distribution of *P. speciosa*. Temperature dominates the distribution of *P. hainanensis*. At present, *P. hainanensis* was only distributed in Hainan Island, which had a tropical monsoon maritime climate with high‐annual temperature and small annual temperature difference, so it was suitable for the growth of *P. hainanensis*. *Polyspora hainanensis* had a narrow distribution area and had been listed as a near‐threatened species in the Red List of Theaceae (Beech et al., 2017). Our prediction results show that the total suitable area of *P. hainanensis* would decrease in the future, but there was a highly suitable area in eastern Taiwan, which can be used as a potential introduction area to expand its population size.

### 4.2 Species differentiation and glacial refugia speculation

**FIGURE 15:**
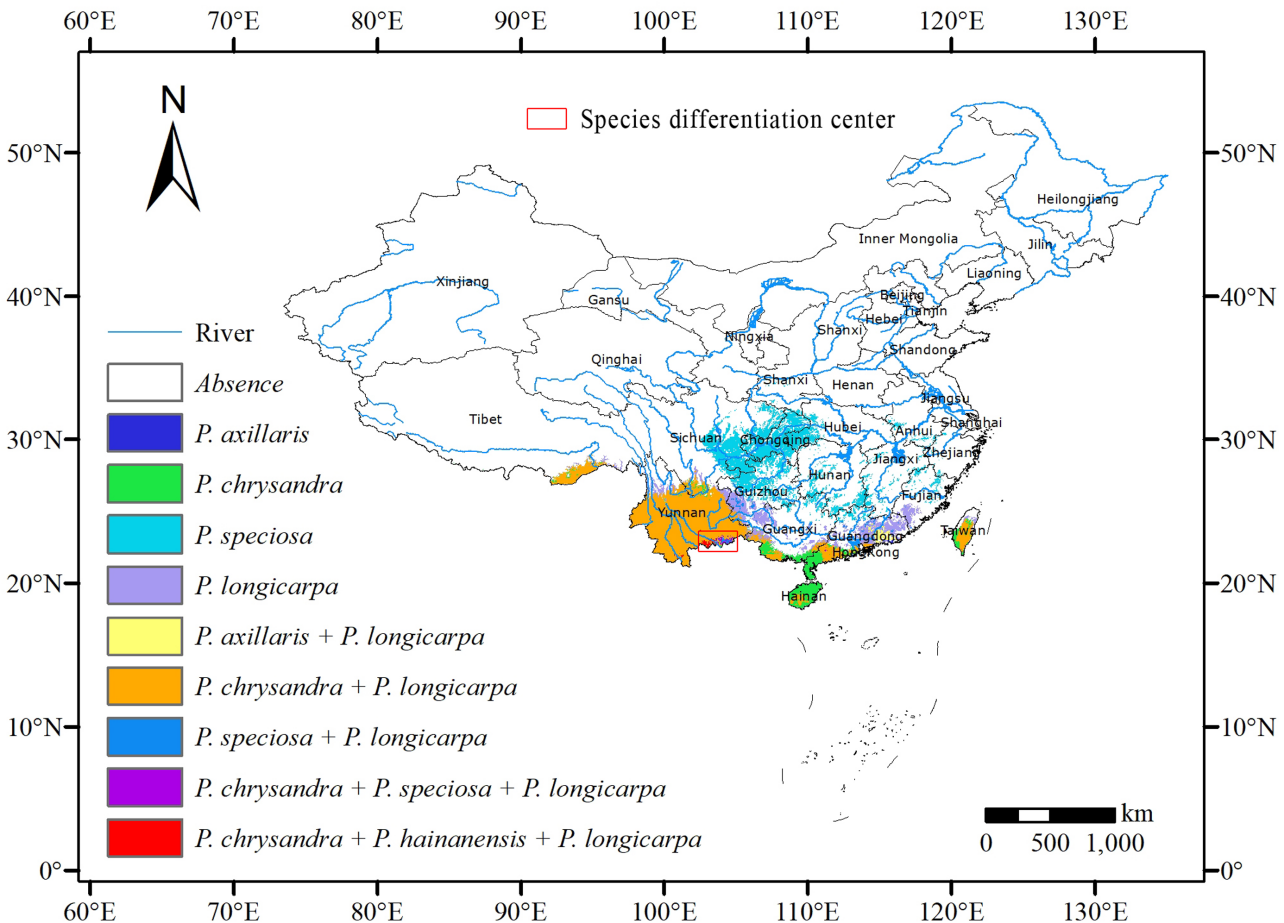
Species differentiation center of Chinese *Polyspora*. Superposition results of the distribution map of five species of *Polyspora* in the last glacial maximum. The simulated distribution maps of each species during the last glacial maximum are the arithmetic average superposed maps of three global climate models. Color blocks indicate independent or overlapping distribution areas of species. The red rectangular box shows the species differentiation center.

In the evolutionary history of species, niche differentiation can promote species differentiation (Zhu et al., 2013), and species distribution models can test the hypotheses in evolutionary biology (Peterson & Nyari, 2008; Ree et al., 2005). Based on the phylogeny and divergence time of the chloroplast genome, species differentiation of genus *Polyspora* began in the late Miocene, and most species completed their differentiation in the late Pliocene (Figure 14). Our estimated differentiation time was consistent with the research results of Yu et al. (2017). Since the Pliocene, the surface temperature had generally declined (Figure 14). During the LGM, some differentiated species stayed in situ or migrated to the common refugia. Figure 15 shows the superposition results of the distribution map of five species of *Polyspora* in the LGM. During the LGM 22,000 years ago, there were overlapping distributions of various types and regions of Chinese *Polyspora* species. Among them, Jinping County, Pingbian County and Maguan County in southeast Yunnan, adjacent to Vietnam, were the common suitable distribution areas for four species of *Polyspora* (*P. chrysandra*, *P. speciosa*, *P. hainanensis*, and *P. longicarpa*), which were located in four clades of the phylogenetic tree (Figure 14). We speculated that this area might be the differentiation center of *Polyspora* species in China.

The LGM was the closest to the human environment in the last 20,000 years, and had a great contrast with the modern climate. At that time, the climate in China was cold and dry, and most areas were grassland and desert. The edge of the grassland could reach the north of the modern evergreen broad‐leaved forest, while the evergreen broad‐leaved forest reached the modern tropical area, and the tropical forest completely disappeared (Li et al., 2018). Figures S34–S38 shows the niche overlaps between the LGM and the current period of *Polyspora* species in China. During the LGM, suitable habitat of *P. axillaris* was mainly located in the Lianhua Mountain area in eastern Guangdong, where might be the glacial refugium of *P. axillaris* (Figure S34). *Polyspora chrysandra* was still widely distributed during the LGM, and the glacial refugium might be located in Ailao Mountain (Figure S35). The glacial refugium of *P. speciosa* might have been located around Chongqing, and expanded to southeast after the glaciation (Figure S36). Due to the disappearance of tropical forests during the LGM, *P. hainanensis* lost its habitat and migrated northwest. Dawei Mountain in southeastern Yunnan might be its glacial refugium (Figure S37). The glacial refugium of *P. longicarpa* might be located in the Gaoligong Mountain of western Yunnan, where it expanded northward in a small range after the glacial period (Figure S38).

The unique topography and climate in southwest China provide rich habitats for animals and plants, making it not only a hotspot of biodiversity in the world, but also an ideal place of species origin and glacial refugia (Gao & Zhao, 2021; Qiu et al., 2011; Tang et al., 2018). By comparing the distribution ranges of various species of *Polyspora* in current and LGM, we found that the main glacial refugia of the genus were located in southwestern China. In addition, Lianhua Mountain in Guangdong Province also formed a relatively independent niche, which weakened the impact of Quaternary climatic turbulence to a certain extent, and became another potential suitable area of *P. axillaris* during the LGM.

### 4.3 Impacts of future climate change on the distribution of *Polyspora*

Climate change is the major factor affecting the large‐scale distribution pattern and migration pattern of species (Pearson & Dawson, 2003; Wu et al., 2021). The prediction results of several climate models show that the surface temperature in China will continue to rise in the future, and the annual precipitation in most parts of the country will also increase. By the end of the century, the average temperature will increase by 2.7–5.4°C, and the annual average precipitation will increase by 17%–30% (Yang, Zhou, et al., 2021). The prediction results showed that the total suitable area of *Polyspora* in China would generally decrease under the four SSPs in the future. Paleoclimatic changes might have profoundly affected the historical spatial and population dynamics of *Polyspora* in ancient China. Our results indicated that an important stable climatic region of *Polyspora* was formed in southern and western Yunnan in ancient China. In the process of future climate change, these areas will remain stable distribution areas of *Polyspora*, which is of great importance for the conservation of this genus. In addition, Taiwan Island, southern Hainan Island and southern Guangxi have relatively stable high‐suitable habitats, and Guangdong Province also has a fragmented and discontinuous stable distribution area. The centroid transfer map shows that in the future, the centroid of *Polyspora* will continue to shift to the southeast by 255 km (SSP585)–340 km (SSP370), and Guangxi may become the distribution center of *Polyspora* in China (Figure 10). Different species within the genus had different responses to future climate change. Suitable habitats of *P. axillaris*, *P. chrysandra*, and *P. speciosa* showed an expansion trend, while *P. hainanensis* and *P. longicarpa* showed a contraction trend. In general, some *Polyspora* species will lose part of their suitable area, but each species has a relatively stable habitat area. Climate change will not cause large‐scale migration or extinction of *Polyspora* species, but reasonable control of carbon emissions will beneficial to the survival and distribution of *Polyspora* species.

## 5 CONCLUSION

In this study, the species geographical distribution data and environmental variables were used to simulate the distribution dynamics of *Polyspora* in China from the LGM to the future. The results showed that the regions south of latitude 25°N in China are suitable for the growth of *Polyspora*. The key climatic factors affecting the distribution of *Polyspora* were precipitation of driest month, temperature seasonality and mean diurnal range. The current potential highly suitable areas of *Polyspora* species were species‐specific, and the potential distribution area of *P. speciosa* was the widest. The main glacial refugia of the genus *Polyspora* in China might be located in the Ailao Mountain, Gaoligong Mountain, Dawei Mountain areas of Yunnan. Jinping County, Pingbian County and Maguan County at the border of China and Vietnam might be the species differentiation center of Chinese *Polyspora*. In the future, moderate climate warming will be conducive to the survival of *P. axillaris*, *P. chrysandra*, and *P. speciosa*. However, climate warming under different SSPs will reduce the suitable habitats of *P. hainanensis* and *P. longicarpa*. The results are helpful for understanding the phylogeography of *Polyspora*, and are of great significance for exploring the responses of plant groups in subtropical evergreen broad‐leaved forests with geological events and climate change in China.

## AUTHOR CONTRIBUTIONS

**Zhi‐Feng Fan:** Conceptualization (equal); data curation (lead); formal analysis (lead); investigation (lead); methodology (lead); resources (lead); software (lead); visualization (lead); writing – original draft (lead). **Bing‐Jiang Zhou:** Methodology (supporting); software (supporting). **Chang‐Le Ma:** Conceptualization (lead); funding acquisition (lead); project administration (lead); supervision (lead); writing – review and editing (equal). **Can Gao:** Data curation (supporting); formal analysis (supporting); investigation (supporting). **Dan‐Ni Han:** Validation (supporting); visualization (supporting). **Yong Chai:** Formal analysis (supporting); methodology (supporting); software (equal).

## FUNDING INFORMATION

This work was financially supported by the Applied Basic Research Project of Yunnan Province, China (2018FG001‐034); Yunnan Postgraduate Adviser Team Construction Project (2019‐101); National Natural Science Foundation of China (31860045).

## CONFLICT OF INTEREST

The authors have no conflict of interest to declare.

## Supporting information


Appendix S1

Supinfo captions

## Data Availability

The data that support the findings of this study are openly available in Dryad at https://doi.org/10.5061/dryad.jq2bvq89x.

## APPENDIX A

### Overview

#### Authorship

Contact: machangle@sina.com

Study link: https://doi.org/10.1002/ece3.9516

#### Model objective

Model objective: Forecast and transfer.

Target output: Habitat suitability maps, continuous occurrence probabilities, and binary maps of potential presence.

#### Focal taxon

Focal taxon: *Polyspora* (Theaceae, Ericales, Embryophyta), six species.

#### Location

Location: China.

#### Scale of analysis

Spatial extent: 73.66, 135.05, 3.86, 53.55 (xmin, xmax, ymin, ymax).

Spatial resolution: 5 × 5 km^2^.

Temporal extent: Pleistocene: Last Glacial Maximum (22,000 BP), mid‐Holocene (6000 BP); Near current (1970–2000); Future (2081–2100).

Temporal resolution: Averages over each period.

Boundary: political.

#### Biodiversity data

Observation type: field survey, GPS tracking, citizen science.

Response data type: presence/absence.

#### Predictors

Predictor types: climatic, edaphic, topographic, habitat.

#### Hypotheses

Hypotheses: Effects of environmental variables (bioclimatic, light, soil, topographic, vegetation) on species distribution and differentiation.

#### Assumptions

Model assumptions: Sampling is adequate, and representative, spatial sampling bias has been successfully corrected. The distribution and differentiation of *Polyspora* were driven by environmental variables. *Polyspora* species are at equilibrium with their environment, their niches expand, contract, or migrate with climate change.

#### Algorithms

Modelling techniques: MaxEnt.

Model complexity: To optimize the model, we used ENMeval package of R software to select modeling parameters: regularization multiplier (RM) and feature classes (FC). The modeling parameters and complexity were different for each species.

Model averaging: Model averaging of 10 replicates.

#### Workflow

Model workflow: (1) Data preparation, environmental variables, and species occurrence data. (2) Data processing, correlation analysis of environmental variables, and data partitioning (training and validation). (3) Model algorithm, modeling parameter selection, and evaluation. (4) Modelling Operation, Simulation of suitable habitat for different species under different periods, different global climate models (GCMs), and different shared socioeconomic pathways (SSPs, future). (5) Generate predictive modeling maps to analyze the niche changes of each species in different periods and different scenarios.

#### Software

Software: MaxEnt version 3.4.4; R (version 4.1.2) with packages corrplot (version 0.92), terra (version 1.5‐12), raster (version 3.5‐15), ENMeval (version 2.0), dplyr (version 1.0.7) and cowplot (version 1.1.1); ArcGIS version 10.2.2.

Data availability: https://doi.org/10.5061/dryad.jq2bvq89x.

### Data

#### Biodiversity data

Taxon names: *Polyspora axillaris*; *Polyspora chrysandra*; *Polyspora hainanensis*; *Polyspora longicarpa*; *Polyspora tiantangensis*; *Polyspora speciosa*.

Taxonomic reference system: Flora of China.

Ecological level: populations, species.

Data sources: National Specimen Information Infrastructure (NSII, http://www.nsii.org.cn); Chinese Virtual Herbarium (CVH, https://www.cvh.ac.cn); Global Biodiversity Information Facility (GBIF, https://www.gbif.org); Literature and field investigation (2020–2022).

Sampling design: Herbarium specimens and opportunistic observations.

Sample size: *Polyspora axillaris* (63); *Polyspora chrysandra* (43); *Polyspora hainanensis* (10); *Polyspora longicarpa* (20); *Polyspora speciosa* (48).

Clipping: We clipped all data to the political boundary of China.

Scaling: Duplicate records in the same localities were removed and spatial autocorrelation was minimized by randomly removing occurrences within 5 km of each other.

Cleaning: We carefully verified each specimen and removed the misidentified occurrence localities.

Absence data: Not applicable.

Background data: We generated 10,000 random background points within the study area.

Errors and biases: We checked each occurrence location coordinate one by one, removed error values, outliers, duplicates, and insufficient information items, so the error rate deemed low.

#### Data partitioning

Training data: We randomly selected 75% of the data for model building and 25% for validation of the predictions.

Validation data: Cross‐validation method.

Test data: Not applicable.

#### Predictor variables

Predictor variables: Climate: annual mean temperature (Bio1, °C), mean diurnal range (Bio2, °C), isothermality (Bio3), temperature seasonality (Bio4), max temperature of warmest month (Bio5, °C), min temperature of coldest month (Bio6, °C), temperature annual range (Bio7, °C), mean temperature of wettest quarter (Bio8, °C), mean temperature of driest quarter (Bio9, °C), mean temperature of warmest quarter (Bio10, °C), mean temperature of coldest quarter (Bio11, °C), annual precipitation (Bio12, mm), precipitation of wettest month (Bio13, mm), precipitation of driest month (Bio14, mm), precipitation seasonality (Bio15), precipitation of wettest quarter (Bio16, mm), precipitation of driest quarter (Bio17, mm), precipitation of warmest quarter (Bio18, mm), precipitation of coldest quarter (Bio19, mm); Topography: elevation (elev, m), Slope (°), Aspect; Soil quality: nutrient availability (sq1), nutrient retention capacity (sq2), rooting conditions (sq3), oxygen availability to roots (sq4), excess salts (sq5), Toxicity (sq6), Workability (sq7, constraining field management); UV‐B radiation: annual mean UV‐B (uvb1, J·m^−2^·day^−1^), UV‐B seasonality (uvb2, J·m^−2^·day^−1^), mean UV‐B of highest month (uvb3, J·m^−2^·day^−1^), mean UV‐B of lowest month (uvb4, J·m^−2^·day^−1^), sum of monthly mean UV‐B during highest quarter (uvb5, J·m^−2^·day^−1^), sum of monthly mean UV‐B during lowest quarter (uvb6, J·m^−2^·day^−1^); Vegetation: 55 vegetation types.

Data sources: Paleoclimatic data were derived from WorldClim version 1.4 dataset (https://www.worldclim.org/data/v1.4/paleo1.4.html); Current, future bioclimatic data and elevation data were derived from WorldClim version 2.1 dataset (https://www.worldclim.org/data/worldclim21.html, https://www.worldclim.org/data/cmip6/cmip6_clim2.5m.html), the slope and aspect data were extracted by the ArcGIS 10.2.2 (http://www.esrichina.com.cn/) spatial analysis function based on the elevation tada; Soil quality data were derived from the Harmonized World Soil Database version 1.2 (HWSD, https://www.fao.org/soils‐portal/data‐hub/soil‐maps‐and‐databases/harmonized‐world‐soil‐database‐v12/zh/); UV‐B radiation data were derived from the gIUV database (https://www.ufz.de/gluv/); Vegetation data attained from the 1:1 million China vegetation dataset, which was derived from the Data Center for Resources and Environmental Sciences, Chinese Academy of Sciences (RESDC, http://www.resdc.cn).

Spatial extent: 73.66, 135.05, 3.86, 53.55 (xmin, xmax, ymin, ymax).

Spatial resolution: Data were collected and prepared at the same resolution as the bioclimatic data: 2.5 min spatial resolution (approximately 5 km).

Coordinate reference system: WGS 1984.

Temporal extent: Not applicable.

Temporal resolution: The raw resolution of bioclimatic data was 2.5 min (approximately 5 km); the raw resolution of gIUV database was 5 arc‐min (approximately 10 km); the raw resolution of HWSD database was 30 arc‐s (approximately 1 km); the raw resolution of the vegetation data was 1 km.

Data processing: For UV‐B, soil and vegetation variables, we used raster package (version 3.5‐15) to unify the resolution consistent with bioclimatic variables.

Errors and biases: Not applicable.

Dimension reduction: Predictor variables were standardized and used correlation analysis to avoid highly correlated variables. We filtered 14–19 environment variables for modeling.

#### Transfer data

Data sources: Bioclimatic variables (https://www.worldclim.org/), version 1.4 for Paleo, version 2.1 for current and future.

Spatial extent: 73.66, 135.05, 3.86, 53.55 (xmin, xmax, ymin, ymax).

Spatial resolution: 5 × 5 km^2^.

Temporal extent: Last Glacial Maximum (22,000 BP), mid‐Holocene (6000 BP); Near current (1970–2000); Future (2081–2100).

Temporal resolution: Averages over each period.

Models and scenarios: We used three GCMs (CCSM4, MIROC‐ESM, and MPI‐ESM‐P) of CMIP5 climate models to simulate the paleo climate scenarios, used four GCMs (CMCC‐ESM2, CNRM‐CM6‐1, EC‐Earth3‐Veg, and MPI‐ESM1‐2‐HR) and four SSPs (SSP126, SSP245, SSP370, and SSP585) of CMIP6 climate models to simulate future climate periods (2081–2100).

Data processing: The paleoclimatic temperature grid layer (bio 1–bio 2, bio 4–bio 11) was uniformly reduced by 10 times using the terra package (version 1.5‐12) to be consistent with the data units (°C) of other periods.

Quantification of novelty: Distance to training data.

### Model

#### Variable pre‐selection

Variable pre‐selection: Ecological pre‐selection of variables we deemed important for the species, down to 14–19 predictors.

#### Multicollinearity

Multicollinearity: Pearson correlation analysis.

#### Model settings

MaxEnt: featureSet (*Polyspora* [linear, quadratic, hinge]).

Model settings (extrapolation): Not applicable.

#### Model estimates

Coefficients: We used the area under the curve (AUC) and continuous Boyce index (CBI) to evaluate model robustness and excluded models for which the AUC was below 0.9 and for which the CBI was below 0.5.

Parameter uncertainty: Cross‐validation was used to determine the optimum tree size yielding the most robust predictions.

Variable importance: Jackknife method in the model.

#### Model selection—model averaging—ensembles

Model selection: Selection of the best individual models based on the AUC value (>0.9) and CBI value (>0.5).

Model averaging: Predicted mean of selected individual models.

Model ensembles: MaxEnt modeling method.

#### Analysis and correction of non‐independence

Spatial autocorrelation: None.

Temporal autocorrelation: None.

Nested data: None.

#### Threshold selection

Threshold selection: The suitability grades of *Polyspora* were divided into four regions: unsuitable region (0–0.1), lowly suitable region (0.1–0.3), moderately suitable region (0.3–0.5), and highly suitable region (0.5–1). In the simulation of centroid transfer, suitable area change, and interspecific overlapping distribution area, the commonly used fixed threshold of 0.5 were used as the critical value of species distribution/nondistribution.

### Assessment

#### Performance statistics

Performance on training data: AUC, BIC.

Performance on validation data: AUC, BIC.

Performance on test data: AUC, BIC.

#### Plausibility check

Response shapes: We checked model plausibility by assessing partial dependence plots.

Expert judgement: Maps of modelled predictions were checked by experts for an ad‐hoc subset of species.

### Prediction

#### Prediction output

Prediction unit: Continuous habitat suitability index (0–1), gain and loss habitat estimation from the binary prediction (presence/absence).

Post‐processing: Projection under past (2 paleo periods × 3 GCMs), current, and future (4 GCMs × 4 SSPs) climate scenarios, 138 different projections in total (one genus and five species, 23 scenario simulation).

#### Uncertainty quantification

Algorithmic uncertainty: Not applicable.

Input data uncertainty: Not applicable.

Parameter uncertainty: Not applicable.

Scenario uncertainty: We compared the differences in species suitable habitats in different GCMs during the same period, focusing on the same distribution areas to reduce the uncertainty in scenarios.

Novel environments: Climate map visualization for each period and scenario.
