# Supplementary material for: Impacts of climate change on species distribution patterns of Polyspora sweet in China
Source: Ecol Evol. 2022 Dec 13;12(12):e9516. doi: 10.1002/ece3.9516 (PMC9747683; doi:10.1002/ece3.9516)
Supplement: Supplementary file 2 — Supinfo captions [file ECE3-12-e9516-s001.docx]

**Supporting Information**

**Article title:** Impacts of Climate Change on Species Distribution Patterns of *Polyspora* Sweet in China

**Authors:** Zhi-Feng Fan, Bing-Jiang Zhou, Chang-Le Ma, Can Gao, Dan-Ni Han, Yong Chai

The following Supporting Information is available for this article:

**Figure S1** Correlation analysis of environmental variables for *Polyspora axillaris.* The lower left part is the correlation value *r*, ranging from -1 to 1, positive values indicate positive correlation, negative values indicate negative correlation, and 0 indicates non correlation. The greater the |*r*|, the stronger the correlation. The upper right part is a graphical transformation of correlation values. Positive correlations are displayed in blue and negative correlations in red color. Color intensity and the size of the circle are proportional to the correlation coefficients (see the right-hand legend).

**Figure S2** Correlation analysis of environmental variables for *Polyspora chrysandra.* The lower left part is the correlation value *r*, ranging from -1 to 1, positive values indicate positive correlation, negative values indicate negative correlation, and 0 indicates non correlation. The greater the |*r*|, the stronger the correlation. The upper right part is a graphical transformation of correlation values. Positive correlations are displayed in blue and negative correlations in red color. Color intensity and the size of the circle are proportional to the correlation coefficients (see the right-hand legend).

**Figure S3** Correlation analysis of environmental variables for *Polyspora speciosa.* The lower left part is the correlation value *r*, ranging from -1 to 1, positive values indicate positive correlation, negative values indicate negative correlation, and 0 indicates non correlation. The greater the |*r*|, the stronger the correlation. The upper right part is a graphical transformation of correlation values. Positive correlations are displayed in blue and negative correlations in red color. Color intensity and the size of the circle are proportional to the correlation coefficients (see the right-hand legend).

**Figure S4** Correlation analysis of environmental variables for *Polyspora hainanensis.* The lower left part is the correlation value *r*, ranging from -1 to 1, positive values indicate positive correlation, negative values indicate negative correlation, and 0 indicates non correlation. The greater the |*r*|, the stronger the correlation. The upper right part is a graphical transformation of correlation values. Positive correlations are displayed in blue and negative correlations in red color. Color intensity and the size of the circle are proportional to the correlation coefficients (see the right-hand legend).

**Figure S5** Correlation analysis of environmental variables for *Polyspora longicarpa.* The lower left part is the correlation value *r*, ranging from -1 to 1, positive values indicate positive correlation, negative values indicate negative correlation, and 0 indicates non correlation. The greater the |*r*|, the stronger the correlation. The upper right part is a graphical transformation of correlation values. Positive correlations are displayed in blue and negative correlations in red color. Color intensity and the size of the circle are proportional to the correlation coefficients (see the right-hand legend).

**Figure S6** Optimization results for MaxEnt mode of *Polyspora axillaris* under different parameter settings. (A) delta.AICc, (B) AUC.train, (C) CBI.train and (D) 10% omission rate, or.10p.avg. Feature classes (H=hinge, L=linear, LQ=linear quadratic, LQH=linear quadratic hinge, LQHP=linear quadratic hinge product, LQHPT= linear quadratic hinge product threshold).

**Figure S7** Optimization results for MaxEnt mode of *Polyspora chrysandra* under different parameter settings. (A) delta.AICc, (B) AUC.train, (C) CBI.train and (D) 10% omission rate, or.10p.avg. Feature classes (H=hinge, L=linear, LQ=linear quadratic, LQH=linear quadratic hinge, LQHP=linear quadratic hinge product, LQHPT= linear quadratic hinge product threshold).

**Figure S8** Optimization results for MaxEnt mode of *Polyspora speciosa* under different parameter settings. (A) delta.AICc, (B) AUC.train, (C) CBI.train and (D) 10% omission rate, or.10p.avg. Feature classes (H=hinge, L=linear, LQ=linear quadratic, LQH=linear quadratic hinge, LQHP=linear quadratic hinge product, LQHPT= linear quadratic hinge product threshold).

**Figure S9** Optimization results for MaxEnt mode of *Polyspora hainanensis* under different parameter settings. (A) delta.AICc, (B) AUC.train, (C) CBI.train and (D) 10% omission rate, or.10p.avg. Feature classes (H=hinge, L=linear, LQ=linear quadratic, LQH=linear quadratic hinge, LQHP=linear quadratic hinge product, LQHPT= linear quadratic hinge product threshold).

**Figure S10** Optimization results for MaxEnt mode of *Polyspora longicarpa* under different parameter settings. (A) delta.AICc, (B) AUC.train, (C) CBI.train and (D) 10% omission rate, or.10p.avg. Feature classes (H=hinge, L=linear, LQ=linear quadratic, LQH=linear quadratic hinge, LQHP=linear quadratic hinge product, LQHPT= linear quadratic hinge product threshold).

**Figure S11** Habitats photographs of *Polyspora* species. (a) *Polyspora axillaris*; (b) *P. chrysandra*; (c)*P. longicarpa*; (d)*P. speciosa*; (e)*P. tiantangensis*; (f) *P. hainanensis*. All the photos were taken by the first author.

**Figure S12** Potential distribution areas of *Polyspora axillaris* under paleoclimate scenarios. Comparison of three global climate models (CCSM4, MIROC-ESM and MPI-ESM-P) in two paleoclimatic periods, the Last Glacial Maximum and mid-Holocene.

**Figure S13** Potential distribution areas of *Polyspora axillaris* under current environmental conditions. When presence probability is < 0.1, unsuitable region; When presence probability is 0.1–0.3, lowly suitable region; When presence probability is 0.3–0.5, moderately suitable region; And when presence probability is > 0.5, highly suitable region.

**Figure S14** Potential distribution areas of *Polyspora axillaris* under future SSP126-SSP245 scenarios. Future projections (2100) are estimated from four global climate models (CMCC‐ESM2, CNRM-CM6-1, EC-Earth3-Veg and MPI-ESM1-2-HR) and two shared socio-economic pathways (SSP126 and SSP245).

**Figure S15** Potential distribution areas of *Polyspora axillaris* under future SSP370-SSP585 scenarios. Future projections (2100) are estimated from four global climate models (CMCC‐ESM2, CNRM-CM6-1, EC-Earth3-Veg and MPI-ESM1-2-HR) and two shared socio-economic pathways (SSP370 and SSP585).

**Figure S16** Potential distribution areas of *Polyspora chrysandra* under paleoclimate scenarios. Comparison of three global climate models (CCSM4, MIROC-ESM and MPI-ESM-P) in two paleoclimatic periods, the Last Glacial Maximum and mid-Holocene.

**Figure S17** Potential distribution areas of *Polyspora chrysandra* under current environmental conditions. When presence probability is < 0.1, unsuitable region; When presence probability is 0.1–0.3, lowly suitable region; When presence probability is 0.3–0.5, moderately suitable region; And when presence probability is > 0.5, highly suitable region.

**Figure S18** Potential distribution areas of *Polyspora chrysandra* under future SSP126-SSP245 scenarios. Future projections (2100) are estimated from four global climate models (CMCC‐ESM2, CNRM-CM6-1, EC-Earth3-Veg and MPI-ESM1-2-HR) and two shared socio-economic pathways (SSP126 and SSP245).

**Figure S19** Potential distribution areas of *Polyspora chrysandra* under future SSP370-SSP585 scenarios. Future projections (2100) are estimated from four global climate models (CMCC‐ESM2, CNRM-CM6-1, EC-Earth3-Veg and MPI-ESM1-2-HR) and two shared socio-economic pathways (SSP370 and SSP585).

**Figure S20** Potential distribution areas of *Polyspora speciosa* under paleoclimate scenarios. Comparison of three global climate models (CCSM4, MIROC-ESM and MPI-ESM-P) in two paleoclimatic periods, the Last Glacial Maximum and mid-Holocene.

**Figure S21** Potential distribution areas of *Polyspora speciosa* under current environmental conditions. When presence probability is < 0.1, unsuitable region; When presence probability is 0.1–0.3, lowly suitable region; When presence probability is 0.3–0.5, moderately suitable region; And when presence probability is > 0.5, highly suitable region.

**Figure S22** Potential distribution areas of *Polyspora speciosa* under future SSP126 scenarios. Future projections (2100) are estimated from four global climate models (CMCC‐ESM2, CNRM-CM6-1, EC-Earth3-Veg and MPI-ESM1-2-HR).

**Figure S23** Potential distribution areas of *Polyspora speciosa* under future SSP245 scenarios. Future projections (2100) are estimated from four global climate models (CMCC‐ESM2, CNRM-CM6-1, EC-Earth3-Veg and MPI-ESM1-2-HR).

**Figure S24** Potential distribution areas of *Polyspora speciosa* under future SSP370 scenarios. Future projections (2100) are estimated from four global climate models (CMCC‐ESM2, CNRM-CM6-1, EC-Earth3-Veg and MPI-ESM1-2-HR).

**Figure S25** Potential distribution areas of *Polyspora speciosa* under future SSP585 scenarios. Future projections (2100) are estimated from four global climate models (CMCC‐ESM2, CNRM-CM6-1, EC-Earth3-Veg and MPI-ESM1-2-HR).

**Figure S26** Potential distribution areas of *Polyspora hainanensis* under current environmental conditions. When presence probability is < 0.1, unsuitable region; When presence probability is 0.1–0.3, lowly suitable region; When presence probability is 0.3–0.5, moderately suitable region; And when presence probability is > 0.5, highly suitable region.

**Figure S27** Potential distribution areas of *Polyspora hainanensis* under paleoclimate scenarios. Comparison of three global climate models (CCSM4, MIROC-ESM and MPI-ESM-P) in two paleoclimatic periods, the Last Glacial Maximum and mid-Holocene.

**Figure S28** Potential distribution areas of *Polyspora hainanensis* under future SSP126-SSP245 scenarios. Future projections (2100) are estimated from four global climate models (CMCC‐ESM2, CNRM-CM6-1, EC-Earth3-Veg and MPI-ESM1-2-HR) and two shared socio-economic pathways (SSP126 and SSP245).

**Figure S29** Potential distribution areas of *Polyspora hainanensis* under future SSP370-SSP585 scenarios. Future projections (2100) are estimated from four global climate models (CMCC‐ESM2, CNRM-CM6-1, EC-Earth3-Veg and MPI-ESM1-2-HR) and two shared socio-economic pathways (SSP370 and SSP585).

**Figure S30** Potential distribution areas of *Polyspora longicarpa* under paleoclimate scenarios. Comparison of three global climate models (CCSM4, MIROC-ESM and MPI-ESM-P) in two paleoclimatic periods, the Last Glacial Maximum and mid-Holocene.

**Figure S31** Potential distribution areas of *Polyspora longicarpa* under current environmental conditions. When presence probability is < 0.1, unsuitable region; When presence probability is 0.1–0.3, lowly suitable region; When presence probability is 0.3–0.5, moderately suitable region; And when presence probability is > 0.5, highly suitable region.

**Figure S32** Potential distribution areas of *Polyspora longicarpa* under future SSP126-SSP245 scenarios. Future projections (2100) are estimated from four global climate models (CMCC‐ESM2, CNRM-CM6-1, EC-Earth3-Veg and MPI-ESM1-2-HR) and two shared socio-economic pathways (SSP126 and SSP245).

**Figure S33** Potential distribution areas of *Polyspora longicarpa* under future SSP370-SSP585 scenarios. Future projections (2100) are estimated from four global climate models (CMCC‐ESM2, CNRM-CM6-1, EC-Earth3-Veg and MPI-ESM1-2-HR) and two shared socio-economic pathways (SSP370 and SSP585).

**Figure S34** Niche overlap map of *Polyspora axillaris* between LGM and current. Simulated distribution map of LGM is the arithmetic average superimposed map of three different global climate models. Green color on the map indicates stable habitat of *Polyspora axillaris* since the LGM. Orange indicates contraction habitat of *Polyspora axillaris* since the LGM. Blue color indicates expansion habitat of *Polyspora axillaris* since the LGM.

**Figure S35** Niche overlap map of *Polyspora chrysandra* between LGM and current. Simulated distribution map of LGM is the arithmetic average superimposed map of three different global climate models. Green color on the map indicates stable habitat of *Polyspora chrysandra* since the LGM. Orange indicates contraction habitat of *Polyspora chrysandra* since the LGM. Blue color indicates expansion habitat of *Polyspora chrysandra* since the LGM.

**Figure S36** Niche overlap map of *Polyspora speciosa* between LGM and current. Simulated distribution map of LGM is the arithmetic average superimposed map of three different global climate models. Green color on the map indicates stable habitat of *Polyspora speciosa* since the LGM. Orange indicates contraction habitat of *Polyspora speciosa* since the LGM. Blue color indicates expansion habitat of *Polyspora speciosa* since the LGM.

**Figure S37** Niche overlap map of *Polyspora hainanensis* between LGM and current. Simulated distribution map of LGM is the arithmetic average superimposed map of three different global climate models. Green color on the map indicates stable habitat of *Polyspora hainanensis* since the LGM. Orange indicates contraction habitat of *Polyspora hainanensis* since the LGM. Blue color indicates expansion habitat of *Polyspora hainanensis* since the LGM.

**Figure S38** Niche overlap map of *Polyspora longicarpa* between LGM and current. Simulated distribution map of LGM is the arithmetic average superimposed map of three different global climate models. Green color on the map indicates stable habitat of *Polyspora longicarpa* since the LGM. Orange indicates contraction habitat of *Polyspora longicarpa* since the LGM. Blue color indicates expansion habitat of *Polyspora longicarpa* since the LGM.

**Appendix A** ODMAP Protocol of Chinese *Polyspora* distribution.

**Table S1** Species occurrence data.

**Table S2** Information of 36 environmental variables.

**Table S3** Environmental factors and modeling parameters for species distribution prediction

**Table S4** Accession numbers of chloroplast genomes used for phylogenetic analyses.

**Table S5** MaxEnt modeling parameters and model accuracy evaluation results of Chinese *Polyspora*.

**Table S6** MaxEnt modeling parameters and model accuracy evaluation results of *Polyspora axillaris*.

**Table S7** MaxEnt modeling parameters and model accuracy evaluation results of *Polyspora chrysandra*.

**Table S8** MaxEnt modeling parameters and model accuracy evaluation results of *Polyspora hainanensis*.

**Table S9** MaxEnt modeling parameters and model accuracy evaluation results of *Polyspora longicarpa*.

**Table S10** MaxEnt modeling parameters and model accuracy evaluation results of *Polyspora speciosa*.

**Table S11** Contribution values for Chinese *Polyspora* species.

**Table S12** Suitable habitats area of *Polyspora* under different periods, different GCMs and different SSPs.

**Table S13** Suitable habitats area of *Polyspora axillaris* under different periods, different GCMs and different SSPs.

**Table S14** Suitable habitats area of *Polyspora chrysandra* under different periods, different GCMs and different SSPs.

**Table S15** Suitable habitats area of *Polyspora speciosa* under different periods, different GCMs and different SSPs.

**Table S16** Suitable habitats area of *Polyspora hainanensis* under different periods, different GCMs and different SSPs.

**Table S17** Suitable habitats area of *Polyspora longicarpa* under different periods, different GCMs and different SSPs.
